# HTS and scRNA-seq revealed that the location and RSS quality of the mammalian TRBV and TRBJ genes impact biased rearrangement

**DOI:** 10.1186/s12864-024-10887-x

**Published:** 2024-10-29

**Authors:** Yingjie Wu, Fengli Wu, Qingqing Ma, Jun Li, Long Ma, Hou Zhou, Yadong Gong, Xinsheng Yao

**Affiliations:** 1 Department of Immunology, Center of Immunomolecular Engineering, Innovation & Practice Base for Graduate Students Education, Zunyi Medical University, Zunyi, China; 2 The Affiliated Taizhou People’s Hospital of Nanjing Medical University, Taizhou, Jiangsu China; 3 Department of Laboratory, The Affiliated Yongchuan Hospital of Chongqing Medical University, Chongqing, China; 4 Department of Central Laboratory, Affiliated guizhou aerospace hospital of Zunyi Medical University, Zunyi City, China

**Keywords:** Mammalian TRB locus, V(D)J rearrangement, Recombination signal sequences, TCR CDR3 repertoire, single-cell RNA sequencing, High-throughput sequencing

## Abstract

The quality of Recombination signal sequences (RSSs), location, and genetics of mammalian V, D, and J genes synergistically affect the recombination frequency of genes; however, the specific regulatory mechanism and efficiency have not been elucidated. By taking advantage of single-cell RNA-sequencing (scRNA-seq) and high-throughput sequencing (HTS) to investigate V(D)J rearrangement characteristics in the CDR3 repertoire, we found that the distal and proximal V genes (or J genes) “to D” gene were involved in rearrangement significantly more frequently than the middle V genes (or J genes) in the TRB locus among various species, including Primates (human and rhesus monkey), Rodentia (BALB/c, C57BL/6, and Kunming mice), Artiodactyla (buffalo), and Chiroptera (*Rhinolophus affinis*). The RSS quality of the V and J genes affected their frequency in rearrangement to varying degrees, especially when the V-RSSs with recombination signal information content (RIC) score < -45 significantly reduced the recombination frequency of the V gene. The V and J genes that were “away from D” had the dual advantages of recombinant structural accessibility and relatively high-quality RSSs, which promoted their preferential utilization in rearrangement. The quality of J-RSSs formed during mammalian evolution was apparently greater than that of V-RSSs, and the D-J distance was obviously shorter than that of V-D, which may be one of the reasons for guaranteeing that the “D-to-J preceding V-to-DJ rule” occurred when rearranged. This study provides a novel perspective on the mechanism and efficiency of V-D-J rearrangement in the mammalian TRB locus, as well as the biased utilization characteristics and application of V and J genes in the initial CDR3 repertoire.

**Supplementary Information:**

The online version contains supplementary material available at 10.1186/s12864-024-10887-x.

## Significance

1. HTS and scRNA-seq were used to sequence the TCRβ CDR3 repertoire of human, rhesus monkey, mouse, bat, and buffalo (central and peripheral tissue samples), revealing non-randomness and non-heterogeneity in the frequency of V and J genes usage, with a consistent trend of biased rearrangement among different mammalian V and J genes.
2. It was first discovered in the TCRβ CDR3 repertoire of Primate (human and rhesus monkey), Rodentia (BALB/c, C57BL/6, and Kunming mice), Artiodactyla (buffalo), and Chiroptera (*Rhinolophus affinis*) in mammals: relative to the location of the D gene in the TRB locus, the recombination frequency of the distal and proximal V genes (or J genes) “to D” gene was obviously higher than that of the middle V genes (or J genes); the RSS quality of V and J genes had important consequences with the participation of V and J genes in rearrangement, especially when V RIC scores < − 45, the efficiency of rearrangement was significantly decreased; in mammalian evolution, the J-RSSs demonstrated a superior level of quality compared with the V-RSSs, and there was a noticeable reduction in the D-J distance compared with the V-D, which determined the order (sequential) and efficiency (bias) of V and J genes involved in rearrangement.

**Supplementary Information:**

The online version contains supplementary material available at 10.1186/s12864-024-10887-x.

## Introduction

The specificity and diversity of mammalian T and B cell receptor (TCR and BCR) are central to the adaptive immune response. In 1959, Burnet proposed the clonal selection theory [1], which suggested that during the development of individual T and B cells, a diverse repertoire of self-tolerance was formed, and clonal expansion after antigen stimulation produced a specific response and formed immunological memory. The random recombination [2] of V (variable), D (diversity), and J (joining) genes on somatic chromosomes with deleting/inserting DNA bases contributed to the diversity of BCR, which was confirmed by Tonerawa et al. in 1976 [3, 4]. To date, only 13 mammalian TCR and BCR VDJC genes have been fully annotated [5]. Immunologists have long been puzzled by the order (sequential) and utilization bias of V(D)J participation in recombination [6–9]. In general, germline V(D)J rearrangement follows the “12/23 rule”, but there are also several special supplementary rules, such as the “beyond 12/23 rule” and “starting D-J recombination (DN1, DN2, DN3) first, then V-DJ recombination (DN3)” [6, 10, 11]. In recent years, a growing number of studies have suggested that V(D)J genes are not involved in random recombination, and their efficiency of rearrangement apparently correlates with multiple factors, such as the genomic position (the physical distance of genes in the locus) [12–14], RSS quality [15–18], and chromatin environment [19].

### The physical position affects the efficiency of V(D)J rearrangement

Mammalian V, D, and J genes are scattered across the locus, and when V(D)J is rearranged, it is necessary to shrink the locus to bring the participating recombinant genes to maximize the potential for rearrangement [20]. As early as 1984, Yancopoulos et al. reported that IGHV close to IGHD was preferentially utilized during pre-B-cell receptor (pre-BCR) rearrangement [12]. Raaphorst et al. reported that TRBJ2-1 is highly frequently utilized in the TCR CDR3 repertoire of the fetal thymus, liver, bone marrow, spleen, and umbilical cord blood, which may be related to its proximity to the D gene in the alpha beta TCR (αβTCR) gene rearrangement [21]. Xiong et al. reported that TRGV at the proximal end of the TRGJ region was dominant in mouse embryonic thymus cells during gamma delta TCR (γδTCR) gene rearrangement [22]. In 2007, Skok et al. demonstrated that the TRB locus of mice was involved in recombination in a loop structure, suggesting that the distal and proximal V (compared with the middle V) may have greater accessibility when recombined with DJ [13]. The compositions of the V, D, and J genes in the TRB locus and their chromosomal locations are highly conserved among different mammals [5]. Therefore, we further speculated that the TRB V(D)J recombinant pattern in mice has the potential to converge consistently across all mammals and plays a dominant role in bias in the initial CDR3 repertoire.

### RSS quality (RIC scores) affects the efficiency of rearrangement

RSS is a noncoding DNA sequence normally consisting of a highly conserved heptamer (CACAGTG), a poorly conserved spacer sequence of 12 or 23 nucleotides (conserved in length but not in sequence), and a conserved nonamer (ACAAAAACC). The RSS of the TCR-β chain is as follows: 3’end of the V gene = 7-23-9, 5’end of the D gene = 7-23-9, and 5’end of the J gene = 9-12-7 [5, 23].

In germline gene rearrangement, the molecule that coordinated V(D)J rearrangement was called the V-D-J recombinase complex, and the discontinuous gene connections involved in rearrangement in the locus depend on the recombinase complex to recognize the RSS. 7-12-9RSS and 9-23-7RSS combine by interacting with a recombinant enzyme complex that specifically recognizes RSS, allowing the rearrangement to follow the “12/23 rule”. The rearrangement process was initiated by the recombination activating gene (RAG) [24, 25], a complex of RAG1 and RAG2 together with a high mobility group (HMG) that recognized the RSS, allowing the association of two different RSSs. The RAG used endonuclease activity to precisely cut a single-stranded notch between the coding region and the RSS, the free 3’-OH group reacted with a phosphodiester bond on the other strand, a hairpin structure was formed at the coding region end, a blunt double-stranded notch was formed at the RSS end, and the two different ends were repaired in a different way. The free 3’-OH group reacted with the phosphodiester bond on the other strand, forming a hairpin structure at the end of the coding region and a blunt double-stranded notch at the end of the RSS, and the two different ends were repaired in different ways [26]. In the coding joint region, the heterodimer Ku70-Ku80 bound and stabilized the DNA ends, then DNA-dependent protein kinase (DNA-PK) and Artemis opened the hairpin, and then the deoxynucleotide terminal transferase (TdT) modified the DNA ends. Finally, DNA ligase IV joined the DNA ends to complete the rearrangement. In the signal joint region, Ku70-Ku80 bound and stabilized the DNA ends, but no further modification was carried out. In the signal joint region, the DNA ends were also stabilized by Ku70-Ku80, but no further modifications were carried out. Instead, DNA ligase IV joined the DNA ends, forming a closed loop independent of the chromosome [27–29]. When the RAG was combined with the RSS, the “accessibility” of the near end can be increased by linear scanning. For the remote end, in addition to rearrangement through “locus contraction”, long-distance rearrangement can also be completed through linear RAG chromatin scanning.

Multiple laboratories have shown that the length of the 12-bp or 23-bp spacer sequence, the conservation of the heptamer and nonamer, and the nucleotide composition at important locations influence the efficiency of RSS in recombination [15]. For example, mutations in the first 3 bases of the heptamer reduced the recombination linking rate; when the base sequence of the last four sites of the heptamer was changed, the efficiency of recombinants in the formation of notches and hairpins was reduced by at least 2 times; for nonamer central site base mutations, such as 9-4G (A to G), the rearrangement frequency decreased to 27%, 9-5 C (A to C) decreased to 10%; for 11 bp RSSs, the rearrangement frequency decreased to 7% of that of the wild type; and for one C-base (13RSSs) added to 12 bp RSSs, the rearrangement frequency reached 11% [15]. In 1994, Akamatsu et al. reported that 11-bp and 13-bp spacer sequences prominently decreased the frequency of rearrangement, whereas when 2 C bases were added to the 14-bp spacer sequence, rearrangement was not detected [30]. We previously analyzed the BCR-H CDR3 repertoire of humans under both healthy and pathological conditions by HTS. Surprisingly, we found that the highly conserved IGHJ4 gene was involved in rearrangements exceeding 40% (significantly higher than those of the other 5 IGHJ genes) [31]. Cowell et al. constructed a statistical model (www.itb.cnr.it/rss/RIC) of quality scores that could calculate RSSs of any length on the basis of plasmid recombinant substrate data by assigning scores from the contribution of each nucleotide in the heptamer, spacer, and nonamer, which has been validated in multiple laboratories [16–18]. For example, when Wu et al. replaced the 3’Vβ31 RSS (RIC score = -37.3) with the 3’Dβ1 RSS (RIC score = -35.2) in transgenic mice, the frequency of recombination increased [32, 33]. However, the mechanism of how to interact with other factors, such as RAG, thus affecting the frequency of use has not been clarified [34].

Studies on RSS quality and binding efficiency of V-D-J recombinase complex have been carried out mainly in specific cell models or in specific transgenic mice. We preliminarily investigated the possible effects of RSS quality (RIC scores) and distance on the frequency of TRBV and TRBJ genes usage involved in recombination during the physiological recombination of five mammals with a large TRB CDR3 repertoire obtained by HTS and scRNA-seq.

### Several other factors affect the efficiency of rearrangement

In 2001, Williams and colleagues reported varying recombination frequencies among genes with the same RSSs, indicating that potential factors may cover the influence of the RSS [35]. Genetic factors [36, 37], epigenetic factors [38], and transcription factors such as Pax5 [39, 40], MHCII E molecule [41], and SNP density [42] all participate in the regulation of the frequency of V(D)J rearrangement.

**Fig. 1:**
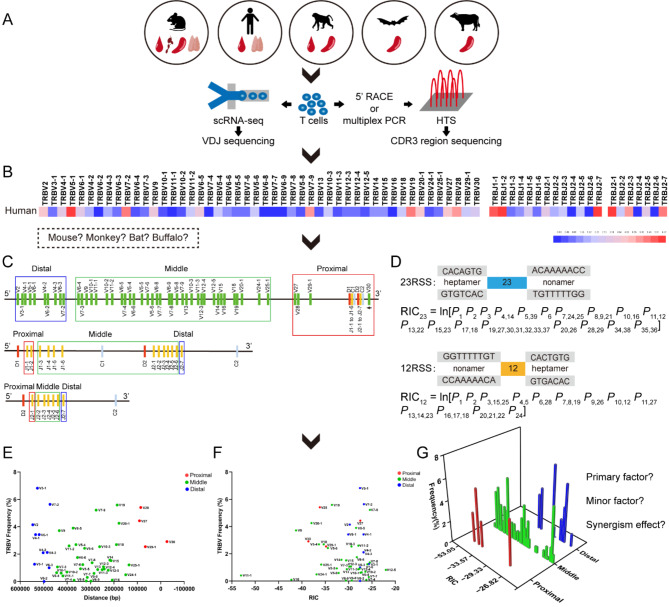
(**A**) Study subjects: humans, rhesus monkeys, BALB/c, C57BL/6, and KM mice, *Rhinolophus affinis*, and buffalo. Study samples: thymus, spleen, peripheral blood and lymph nodes. Sequencing technology: HTS TCR CDR3 and scRNA-TCR CDR3. (**B**) Heatmap of the frequency of VDJ genes usage of unique CDR3 sequences in the TCRβ CDR3 repertoires (human sample example). (**C**) Distal, middle, and proximal V genes ‘‘to D’’ gene and distal, middle, and proximal J genes ‘‘to D’’ gene in the TRB locus (human TRB example). (**D**) Principle of the RIC scores (The RSS normative quality scoring criteria (RIC_23_ = In[*P*_1_*P*_2_*P*_3_*P*_4,14_*P*_5,39_*P*_*6*_*P*_*7,24,25*_*P*_8,9,21_*P*_10,16_*P*_11,12_*P*_13,22_*P*_15,23_*P*_17,18_*P*_19,27,30,31,32,33,37_*P*_20,26_*P*_28,29_*P*_34,38_*P*_35,36_] and RIC_12_ = In[*P*_1_*P*_2_*P*_3,15,25_*P*_4,5_*P*_6,28_*P*_7,8,19_*P*_9,26_*P*_10,12_*P*_11,27_*P*_13,14,23_*P*_16,17,18_*P*_20,21,22_*P*_24_])). (**E**) Effect of distance on V or J rearrangement efficiency (human sample example). (**F**) Effect of RSS quality on V or J rearrangement efficiency (human sample example). (**G**) Priorities of V or J recombination efficiency of distance and RSS synergy (human sample example)

Currently, research on the efficiency of V(D)J rearrangement and the contributions of different factors to specific cell models and transgenic mouse models is very limited. We utilized HTS and scRNA-seq to sequence the TCRβ CDR3 repertoire from central and peripheral tissue samples from humans, rhesus monkeys, mice, bats, and buffaloes. Our analysis focused on the frequency of V and J gene subfamily usage and its possible relationship with RSS quality (RSS scores) and the distance from the D gene (Fig. 1), providing a new perspective for the study of the mechanisms, efficiency, and applications of mammalian VDJ biased rearrangement.

## Materials and methods

### Sample collection

The HTS and scRNA-seq data for human samples were obtained from the ENA (accession number: PRJEB41936) and NCBI (accession number: GSE168859) databases. The HTS and scRNA-seq data for the rhesus monkey samples were downloaded from the NCBI database (accession numbers: PRJNA389234 and PRJNA746267). The scRNA-seq data for C57BL/6 mouse samples were obtained from the NCBI database (accession number: GSE168944). Our study did not involve humans, rhesus monkeys, or C57BL/6 mice. We only used data from public databases.

All experimental procedures conducted in our group were performed under the guidelines of animal welfare guidelines. BALB/c and Kunming mouse samples (HTS) were obtained from the Animal Experiment Center of Zunyi Medical University. Bat samples (HTS) were collected from Xishui County, Zunyi City, Guizhou Province, China, by a specialized capture team from Guizhou Normal University. Both the mice and bats were euthanized by cervical dislocation. Buffalo samples (HTS) were sourced from an abattoir that followed all the ethical standards of animal slaughter in Nanning City, Guangxi Province, China. The buffalo samples were approved for research purposes and successfully passed local health and veterinary inspections. The collection of bat, buffalo, and mouse samples was approved by the Ethics Committee of Zunyi Medical University (the bat and mouse project were approved under permit number (2018)2-261, and the buffalo project was approved under permit number ZMU21-2203-111).

### TCRβ CDR3 repertoire sequencing data

The HTS data from human blood and thymus samples were obtained from the ENA database (accession number: PRJEB41936). Four blood samples (ERZ1694578, ERZ1694579, ERZ1694580, and ERZ1694581) and 4 thymus samples (ERZ1694549, ERZ1694551, ERZ1694560, and ERZ1694569) were obtained from 4 infants aged 7, 52, 107 and 156 days, respectively. After the samples were processed, DNA was extracted from PBMCs and thymic cells and sequenced via the ImmunoSEQ™ assay and the Illumina platform [43].

The scRNA-seq data from human blood samples were stored in the NCBI database (accession number: GSE168859). Blood samples (GSM171626, GSM171627, GSM5171634, GSM171635, and GSM171642) were collected from five healthy blood donors aged 39, 71, 55, 68 and 41 years. Density gradient centrifugation was used to isolate the PBMCs. Sequencing was performed on the Illumina HiSeq 3000 platform [44].

We selected tissue samples from the spleen (SRR22437999, SRR22437998, SRR22437997) and thymus (SRR22438002, SRR22438001, and SRR22438000) of 6 2-month-old female BALB/c mice and thymus samples from 3 Kunming (KM) mice (SRR24908413, SRR24908412, and SRR24908411). Total RNA was extracted, and a TCRβ CDR3 repertoire was constructed by the 5’RACE method. Sequencing was performed on the Illumina NovaSeq 6000 platform. The HTS data from the mice have been uploaded to the NCBI database (accession numbers: PRJNA906203 and PRJNA982279).

The scRNA-seq data from peripheral tissue samples of C57BL/6 mice were obtained from the NCBI database (accession number: GSE168944). Total T cells in peripheral tissue samples from lymph samples (GSM5172690, GSM5172691, and GSM5172698), spleen samples (GSM5172688, GSM5172689, and GSM5172696) and blood samples (GSM5172686, GSM5172687, and GSM5172694) were isolated from 3 C57BL/6 mice aged 6-8 weeks. Total T cells from three peripheral tissue samples were pre-enriched by immunomagnetic negative selection to prepare cDNA repertoire, followed by single-cell sequencing. Sequencing was completed on the Illumina HiSeq 4000 platform [45].

The HTS data from rhesus monkey blood samples were downloaded from the NCBI database (accession number: PRJNA389234). A blood sample from a rhesus monkey (SRR5647486) was collected from a healthy 5-year-old female Chinese rhesus monkey. PBMCs were separated by density centrifugation, and total RNA was extracted. The TCRβ CDR3 repertoire was constructed via the 5’RACE method. Sequencing was conducted on the Illumina HiSeq 2000 platform [46].

The scRNA-seq data from peripheral tissue samples from rhesus monkeys were stored in the NCBI database (accession number: PRJNA746267). The T cells in the peripheral tissues of rhesus monkeys were PBMCs (SRR15249806 and SRR15249810), splenocytes (SRR15249798) and FACS-sorted stimulus non-proliferative T cells (SRR15249814) and proliferating T cells (SRR15249812). After cDNA amplification, the V(D)J repertoire was constructed. Sequencing was performed on the Illumina NextSeq 500 platform [47].

We collected spleen samples from three *Rhinolophus affinis* (SRR21464510, SRR21464509, and SRR21464508) and extracted total RNA. Sequencing was performed on the Illumina HiSeq 1500 platform after the TCRβ CDR3 repertoire was prepared by the 5’RACE method. The HTS data have been uploaded to the NCBI database (accession number: PRJNA877449).

We collected spleen samples from six buffaloes (SRR22523497, SRR24889447, SRR24889446, SRR24889445, SRR24889444, and SRR24889443) and then extracted total DNA to construct a TCRβ CDR3 repertoire by multiplex PCR. Sequencing was performed on the MGISEQ-2000RS platform. The HTS data have been shared with the NCBI database (accession numbers: PRJNA908273 and PRJNA982388).

All sequencing data can be downloaded through the accession numbers in Supplementary Tables 1 to 3. The data source for humans, mice, rhesus monkeys, and buffaloes in this study was the same as that for another study by our research group [48].

### Rearrangement and the frequency of V and J genes usage in the TCRβ CDR3 repertoires

MiXCR (Version 3.0.13) [49] was used to analyze raw data from humans, mice, rhesus monkeys, bats, and buffaloes. The JSON format background reference library of human, mouse, and rhesus monkey in MiXCR was based on the TRB VDJC reference sequence provided by IMGT (https://www.imgt.org/). Moreover, for bat and buffalo, the VDJC background reference library was derived from the TRB VDJC annotation by our research group. Data filtering of MiXCR output: (1) deleted the Unproductive sequences containing “_” and “*”, and saved only the Productive Clonotype sequences; (2) further filtered the Productive Clonotype sequences, removing sequences in the CDR3 region amino acid sequence that did not start with “C” and end with “F”; and (3) deleted the TRBV and TRBJ sequences of the ORF and Pseudogenes. This study analyzed the Productive Clonotype sequences of the functional V and J genes. ORF, P and F were defined according to IMGT functionality (https://www.imgt.org/IMGTScientificChart/SequenceDescription/IMGTfunctionality.html); (4) statistical analysis of the rearrangement of V and J genes in the recombination pattern of V_X_-D1-J1, V_X_-D1-J2, and V_X_-D2-J2 in the Productive Clonotype sequences. Data analysis and visualization were performed with GraphPad Prism software (Version 9.3), Heml Data analysis software and OriginPro software (Version 2023). The Unique Clonotypes of TCRβ CDR3 of 5 species are shown in Supplementary Tables 1 to 3. The frequencies of the V and J genes in all the functional sequences of the 5 species are included in Supplementary Tables 4 to 8.

### Divided V and J genes from distal, middle, and proximal

**Fig. 2:**
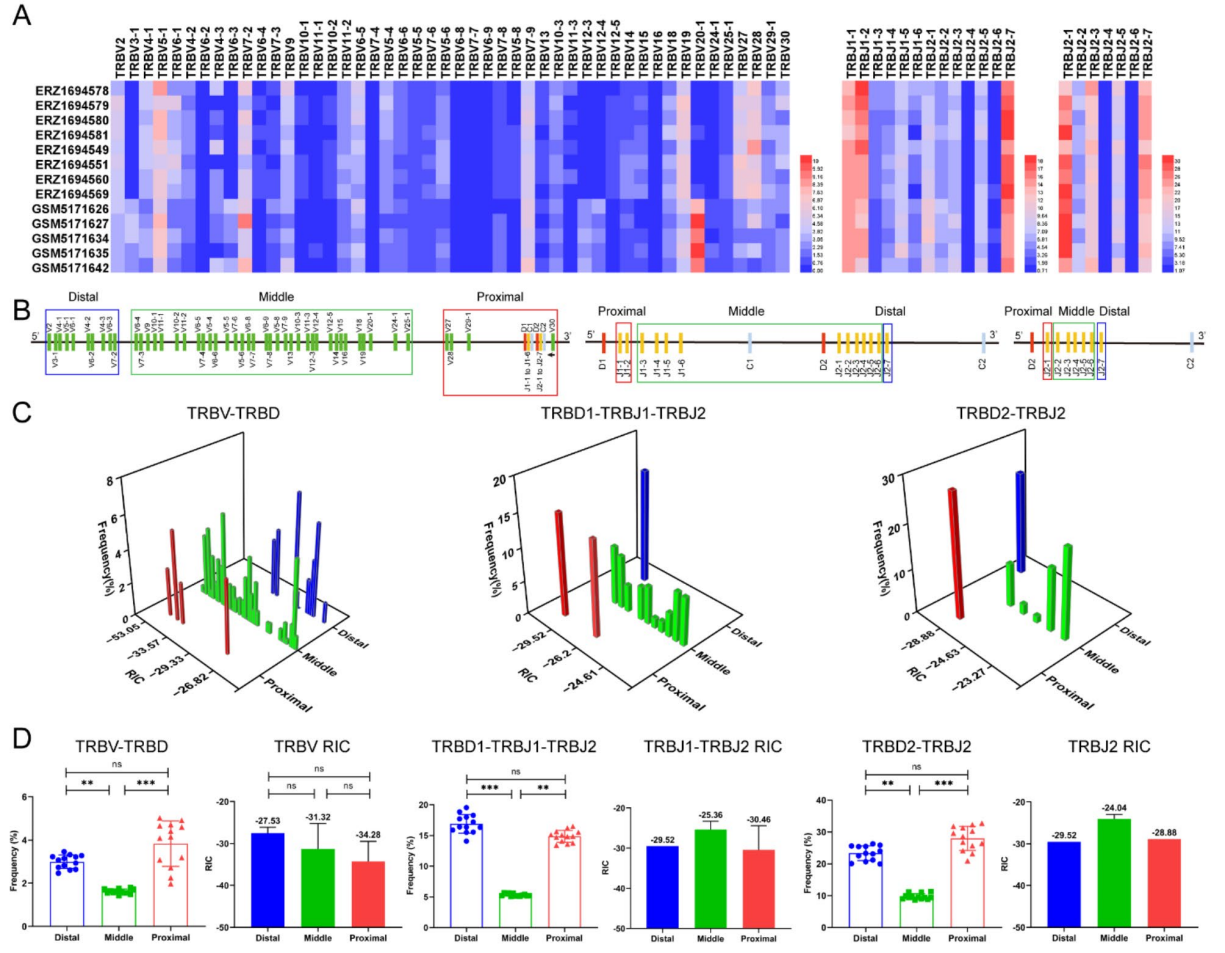
The frequency of V and J genes usage correlations with distance and RSS in the TCRβ CDR3 repertoires of 13 human samples. (**A**) Heatmap of the frequency of V and J genes usage. (**B**) Distal, middle, and proximal gene families of V and J genes ‘‘to D’’ gene. (**C**) Three-dimensional analysis plot of the frequency V or J genes usage in the distal, middle, and proximal groups and RIC scores. (**D**) Statistical comparative analysis of the frequency V or J genes usage in the distal, middle, and proximal groups and RIC scores

**Fig. 3:**
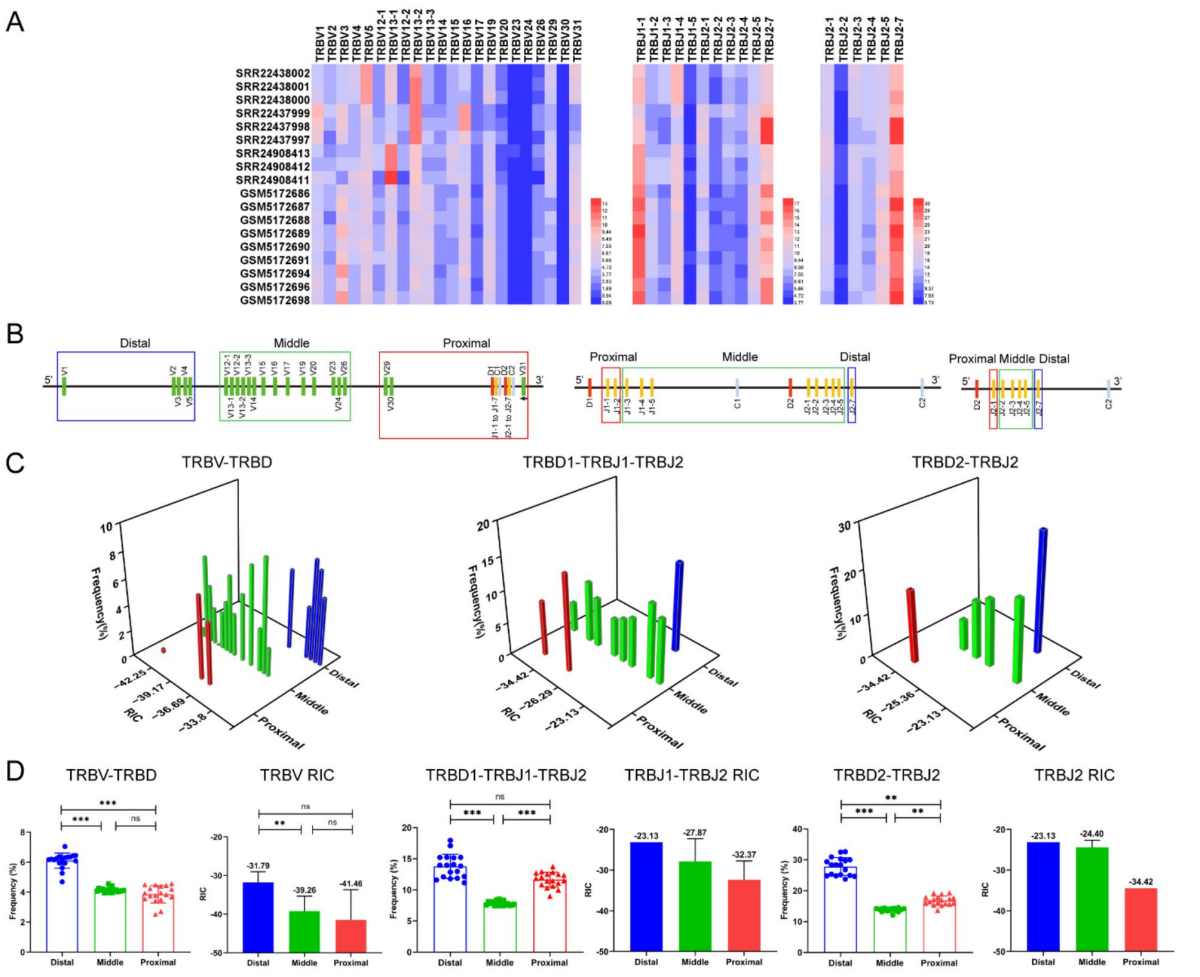
The frequency of V and J genes usage correlations with distance and RSS in the TCRβ CDR3 repertoires of 18 mouse samples. (**A**) Heatmap of the frequency of V and J genes usage. (**B**) Distal, middle, and proximal gene families of V and J genes ‘‘to D’’ gene. (**C**) Three-dimensional analysis plot of V or J usage frequency in the distal, middle, and proximal groups and RIC scores. (**D**) Statistical comparative analysis of V or J usage frequency in the distal, middle, and proximal groups and RIC scores

**Fig. 4:**
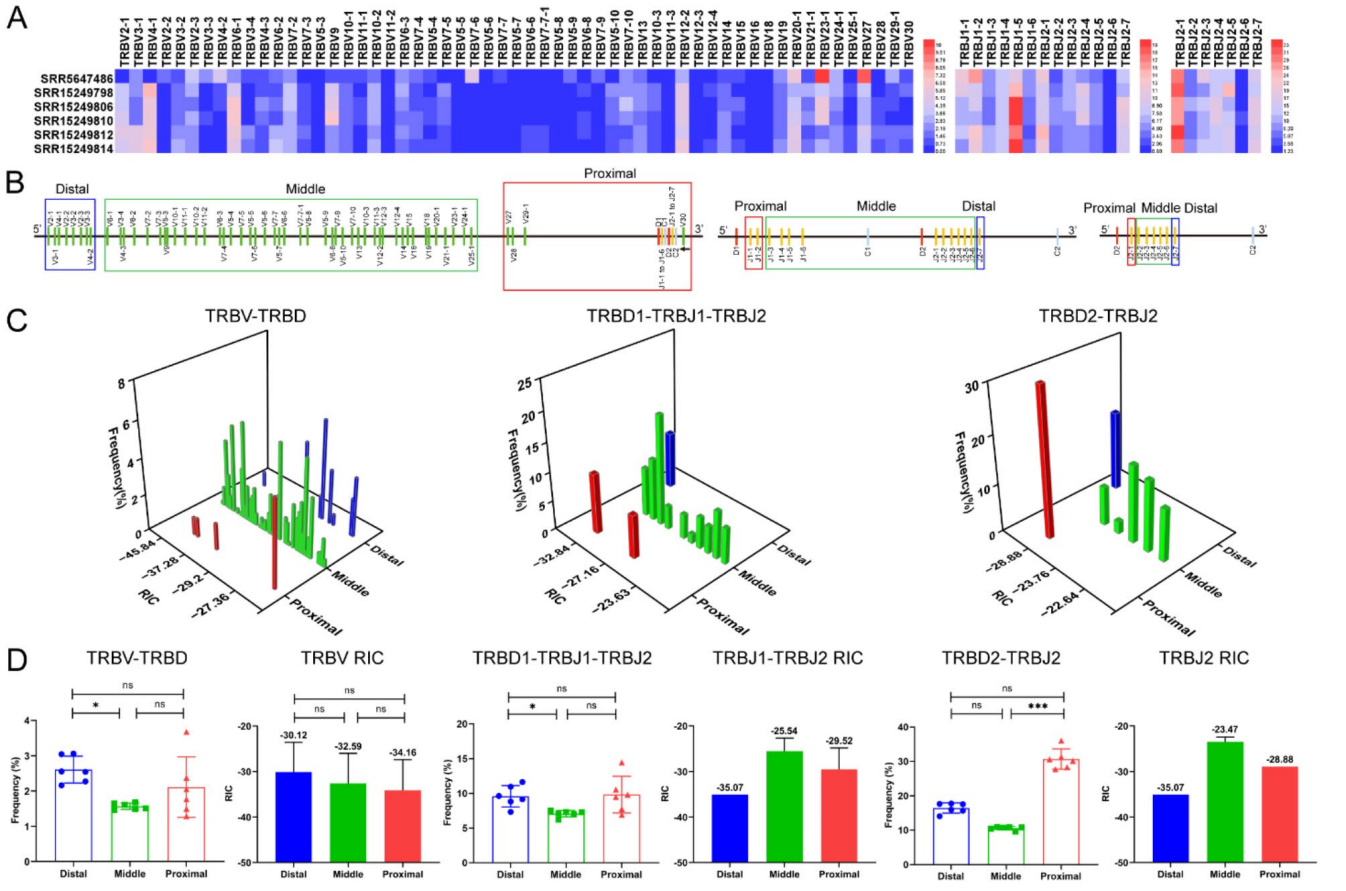
The frequency of V and J genes usage correlations with distance and RSS in the TCRβ CDR3 repertoires of 6 monkey samples. (**A**) Heatmap of the frequency of V and J genes usage. (**B**) Distal, middle, and proximal gene families of V and J genes ‘‘to D’’ gene. (**C**) Three-dimensional analysis plot of V or J usage frequency in the distal, middle, and proximal groups and RIC scores. (**D**) Statistical comparative analysis of V or J usage frequency in the distal, middle, and proximal groups and RIC scores

**Fig. 5:**
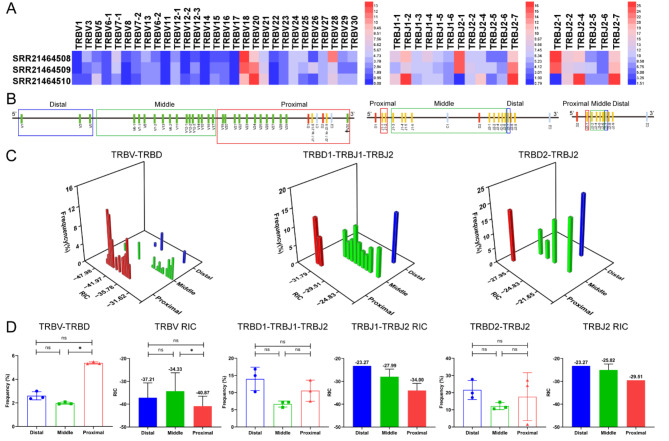
The frequency of V and J genes usage correlations with distance and RSS in the TCRβ CDR3 repertoires of 3 bat samples. (**A**) Heatmap of the frequency of V and J genes usage. (**B**) Distal, middle, and proximal gene families of V and J genes ‘‘to D’’ gene. (**C**) Three-dimensional analysis plot of V or J usage frequency in the distal, middle, and proximal groups and RIC scores. (**D**) Statistical comparative analysis of V or J usage frequency in the distal, middle, and proximal groups and RIC scores

**Fig. 6:**
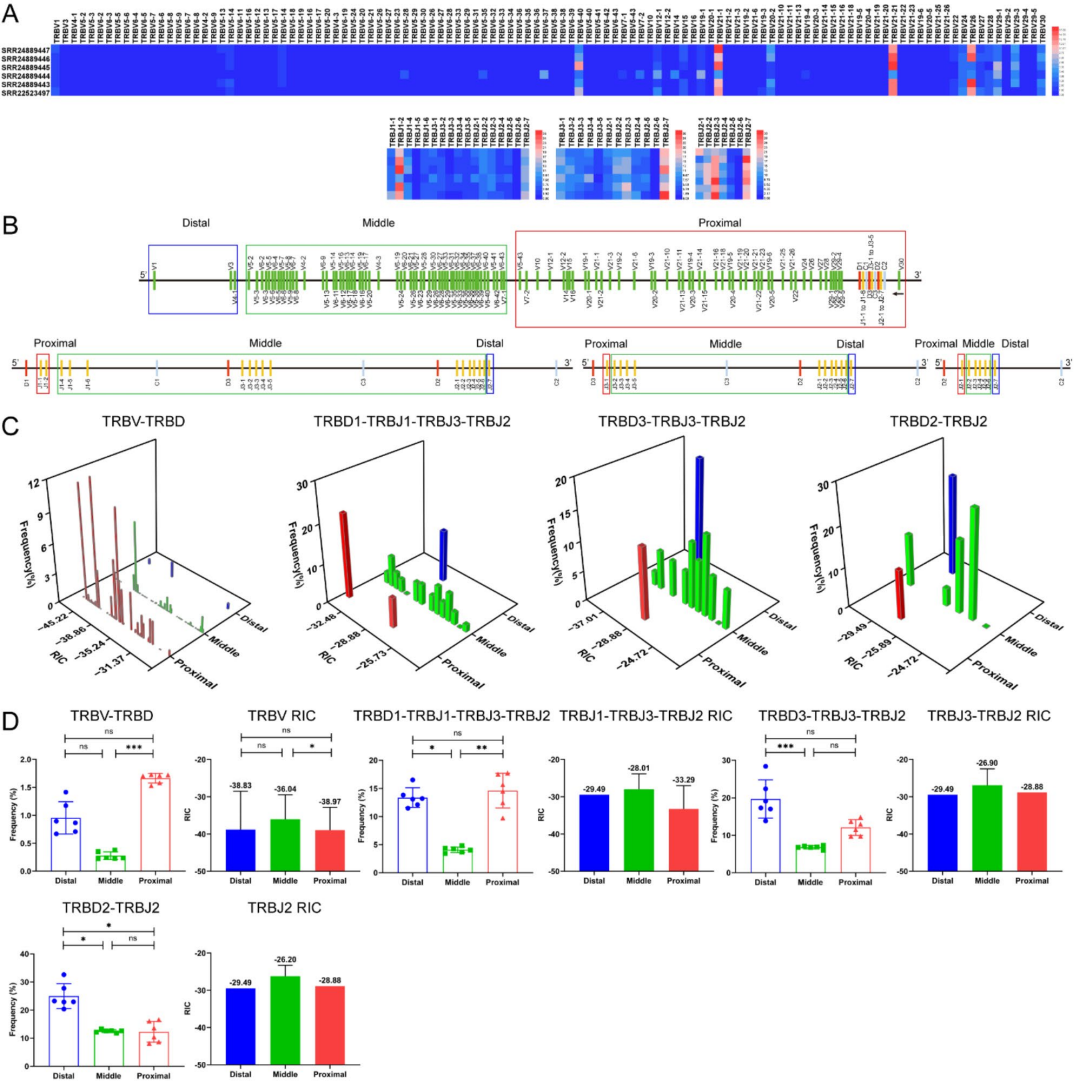
The frequency of V and J genes usage correlations with distance and RSS in the TCRβ CDR3 repertoires of 6 buffalo samples. (**A**) Heatmap of the frequency of V and J genes usage. (**B**) Distal, middle, and proximal gene families of V and J genes ‘‘to D’’ gene. (**C**) Three-dimensional analysis plot of V or J usage frequency in the distal, middle, and proximal groups and RIC scores. (**D**) Statistical comparative analysis of V or J usage frequency in the distal, middle, and proximal groups and RIC scores

Schematic maps of the TRB locus (Figs. 2, 3, 4, 5 and 6) from humans, mice, rhesus monkeys, bats, and buffaloes were created using the physical distances of the functional VDJC gene in the TRB locus obtained from IMGT. Our research group annotated the TRB locus of both bats and buffaloes [50, 51]. On the basis of the distance and position of the 30 V gene families in the TRBV locus across multiple species, including human, mouse, rhesus monkey, bat and buffalo, the genes were separated into three groups categorized as “distal, middle, and proximal” according to their relatively large value of spacing. In the recombinant analysis of D1-J, on the basis of the location of the 13 J genes, the closest and least spaced genes J1-1 and J1-2 were categorized as proximal, TRBJ2-7 was classified as distal, and the remaining genes were considered as middle. J2-1 was the proximal, J2-7 was the distal, and the rest were the middle. In the recombinant analysis of D2-J2, J2-1 was the proximal, J2-7 was the distal, and the rest were the middle. The actual physical distances of V-D and D-J and the rules for grouping them by distance are shown in Supplementary Tables 4 to 8.

### RSSs quality (RIC scores)

The RSS of each VDJ gene in 13 mammals was evaluated on the basis of the quality scoring standard established by Cowell et al. [17]. The scoring system involved assessing the impact of individual nucleotides in the heptamer, spacer, and nonamer. The current RIC score ranges from -1000 (very poor) to 0 (very good). The available experimental results suggested that TRBV genes with RIC scores between -45 and -58 were either unable to participate in rearrangements or rearranged at levels below the average frequency [14, 16–18, 52, 53]. Statistical models were utilized to determine the theoretical potential for rearrangements of RSSs. The algorithm was capable of computing the information score of signal sequences of varying lengths, predicting the quality of 12RSSs and 23RSSs, and analyzing the impact of RSS quality on VDJ genes usage during rearrangement. The Recombination Signal Sequences Site (https://www.itb.cnr.it/rss) contained only humans and mice genome-wide cRSS sequences. We performed calculations for five mammals via both human and mouse RIC computational models, and the RIC results obtained from the two outputs were similar. We used the mouse RIC calculation model to calculate mice RIC scores; for humans, monkeys, bats, and buffaloes RIC scores, we uniformly used the humans RIC calculation model [14, 16–18, 32, 33]. The RIC scores for all functional V, D, and J genes are listed in Supplementary Tables 4 to 8. According to the literature [16], functional V RIC score below -45 was classified as a very low RIC score.

### Statistical analysis

Kruskal-Wallis H Test and Mann Whitney U Test were used to perform differential analyses for each sample and group of samples. *P* value < 0.05 was statistically significant (*P value < 0.05, **P value < 0.01, ***P value < 0.001).

## Results

### Basic information for each sample and unique clonotypes of the TCRβ CDR3 repertoire

Details of the mammals, tissues, starting materials (DNA or RNA), library preparation approaches, sequencing technologies, total unique CDR3 clonotypes, and accession number for each sample are listed in Supplementary Tables 1 to 3. Primates (human samples = 13, Supplementary Table 1; rhesus monkey samples = 6, Supplementary Table 3), Rodentia (BALB/c mouse samples = 6; C57BL/6 mouse samples = 9; KM mouse samples = 3, Supplementary Table 2), Artiodactyla (buffalo samples = 6, Supplementary Table 3), and Chiroptera (*Rhinolophus affinis* samples = 3, Supplementary Table 3) were used. The HTS or scRNA-seq TCRβ CDR3 for each sample was submitted to public database, which can be found by the accession number. Each scRNA-seq sample contained approximately 5000 CDR3 sequences, whereas each HTS sample had over 10,000 CDR3 sequences. One bat sample, however, had only 1770 sequences. The number of CDR3 sequences was consistent with the statistical analysis requirements for the frequency of V or J gene usage.

### Comparative analysis of the frequency of V and J genes usage of CDR3 repertoires between HTS and scRNA-seq

In this study, the research samples of the humans TRB CDR3 repertoires included 8 HTS samples and 5 scRNA-seq samples (Supplementary Table 1, Fig. 2), the research samples of the mice TRB CDR3 repertoires included 9 HTS samples and 9 scRNA-seq samples (Supplementary Table 2, Fig. 3), and the research samples of the rhesus monkeys TRB CDR3 repertoires included 1 HTS sample and 5 scRNA-seq samples (Supplementary Table 3, Fig. 4). The research samples of the bats TRB CDR3 repertoires included 3 HTS samples (Supplementary Table 3, Fig. 5), and the research samples of the buffaloes TRB CDR3 repertoires included 6 HTS samples (Supplementary Table 3, Fig. 6). The results revealed that the number of TRB CDR3 clonotypes in HTS samples was higher than that in scRNA-seq samples, especially in mice. The number of mice CDR3 clonotypes in HTS (5’RACE) samples was significantly higher than that in scRNA-seq samples. The number of mice TRB CDR3 clonotypes in the HTS samples constructed by 5’RACE method ranged from 416,140 to 846,904, which was significantly higher than that in the scRNA-seq samples, which ranged from 2777 to 10,158. However, the frequency of V and J genes usage in the HTS samples were almost identical to those in the scRNA-seq samples, and only some differences existed in the frequency of V and J genes usage, such as TRBV3-1 and TRBV20-1 in humans (Fig. 2A), TRBV3 in mice (Fig. 3A), and TRBV2-1 and TRBV4-1 in rhesus monkeys (Fig. 4A). These results indicate that the distance effect on the frequency of V and J genes usage was generally consistent in both the HTS and scRNA-seq CDR3 repertoires (Figs. 2A, 3A and 4A).

### Distance between V (or J) genes and D gene, RIC scores of V and J genes, and recombination frequency of V and J genes in different mammals

On the basis of the location of the D gene in the mammalian TRB locus, the V or J genes were divided into distal, middle, and proximal regions. The RIC scores of each V and J gene and the frequency of the unique sequences accounting for TCRβ CDR3 are shown in Supplementary Tables 4 to 8: human (Supplementary Table 4), mouse (Supplementary Table 5), rhesus monkey (Supplementary Table 6), bat (Supplementary Table 7), and buffalo (Supplementary Table 8). Each sample in this study demonstrated a biased use of V and J genes. In different tissue samples of each mammal, the frequency of V and J genes usage was essentially the same, and the frequency of V and J genes in each subfamily of the five mammals was consistent (Supplementary Fig. 1).

### The position of TRB V or J genes and the effect of RIC scores on the rearrangement frequency of V or J genes

For the V and J genes of each mammal shown in Figs. 2, 3, 4, 5 and 6, “A” shows the frequency of use through heatmaps, “B” shows the groups of V and J genes on the basis of the distance between V-to-D and D-to-J, “C” shows the synergistic effect of V or J gene usage, and “D” shows the statistical analysis between frequency of use and distance as well as that between frequency of use and RIC. The relationships between the frequency of use of all V and J genes and the specific physical location of each mammal are shown in Supplementary Fig. 2.

Table 1 summarizes the relationships among the frequency of V and J genes usage, the groups based on divided distance, and the RIC scores in each mammal, and the main characteristics are as follows: (1) the distal and proximal V and J were used significantly more frequently than the middle; (2) overall, the middle V and J had the highest RIC scores but the lowest frequency of use; (3) the RIC scores of the distal V and J were generally higher than the proximal, and the frequency of use was higher than the proximal; (4) the RIC scores of the proximal V and J were the lowest overall, but the frequency was significantly higher than the middle; (5) the RIC score of proximal V30 was -50.42, and the frequency of use was only 0.33%, which led to a significant decrease in the average usage of the proximal V (V29, V30, and V31) in mice.

**Table 1:** The relationship among the frequency of V and J genes usage, the groups based on divided distance, and the RIC scores in each mammal

	V		D1-J		D2-J2		D3-J	
	Frequency	V RIC score	Frequency	J RIC score	Frequency	J RIC score	Frequency	J RIC score
Human (Fig. 1**)**	P **>** D **>** M	D **>** M **>** P	D **>** P **>** M	M **>** D **>** P	P **>** D **>** M	M **>** P **>** D		
Mouse (Fig. 2**)**	*D* ***>*** *M* ***>*** *P*	*D* ***>*** *M* ***>*** *P*	*D* **>** P **>** M	*D* **>** M **>** P	*D* **>** P **>** M	*D* **>** M **>** P		
Monkey (Fig. 3**)**	*D* **>** P **>** M	*D* **>** M **>** P	P **>** D **>** M	M **>** P **>** D	P **>** D **>** M	M **>** P **>** D		
Bat (Fig. 4**)**	P **>** D **>** M	M **>** D **>** P	*D* **>** P **>** M	*D* **>** M **>** P	*D* **>** P **>** M	*D* **>** M **>** P		
Buffalo (Fig. 5**)**	P **>** D **>** M	M **>** D **>** P	P **>** D **>** M	M **>** D **>** P	D **>** P **>** M	M **>** P **>** D	D **>** P **>** M	M **>** P **>** D

*Note* (1) D: Distal region, M: Middle region, P: Proximal region. (2) The frequency of D, M, and P of V genes in mice was consistent with the V RIC scores, with D > M > P; the frequency of J genes in mice and J genes in bats was consistent with the J RIC scores; and the frequency of V genes of D in monkeys was consistent with the V RIC. (3) Distance was the main influence on the frequency of V and J genes usage, with P and D dominating over M; the RIC score was related to the frequency of V and J genes usage, and very low RIC scores of V genes affected distance

**Fig. 7:**
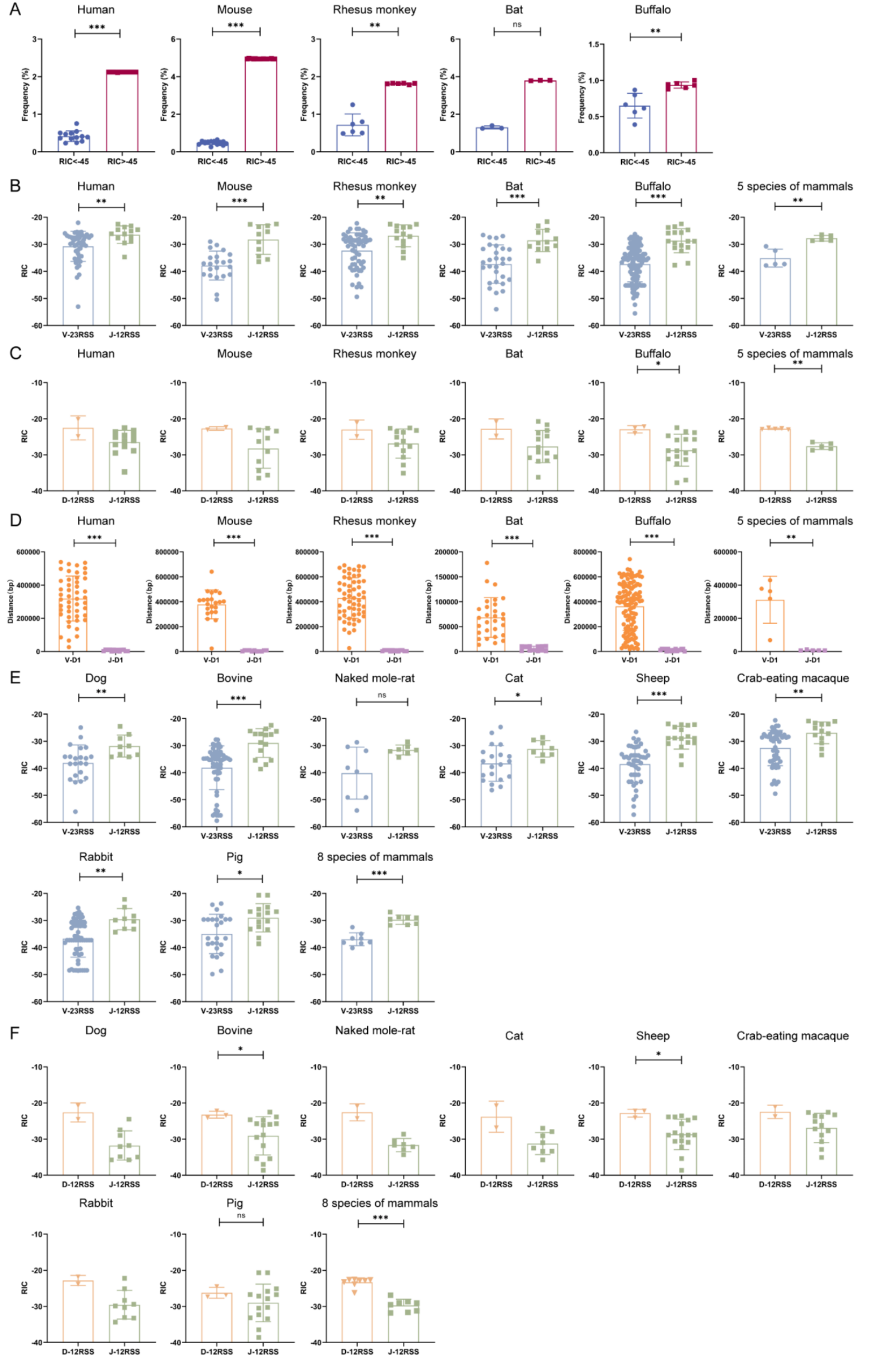
RIC scores and distance analysis of the efficiency (bias) and sequence (sequential) of V and J genes involved in rearrangement in humans, mice, rhesus monkeys, bats, and buffaloes. (**A**) Correlations between recombination frequency and RIC scores below -45 in humans, mice, rhesus monkeys, bats, and buffaloes. (**B**) Comparative analysis of V-23RSS RIC scores and J-12RSS RIC scores in humans, mice, rhesus monkeys, bats, and buffaloes. (**C**) Statistical comparative analysis of 5’D-12RSS RIC scores and 5’J-12RSS RIC scores in humans, mice, rhesus monkeys, bats, and buffaloes. (**D**) Comparative analysis of the V-D distance and J-D distance of humans, mice, rhesus monkeys, bats, and buffaloes. (**E**) Comparative analysis of V-23RSS RIC scores and J-12RSS RIC scores for 8 other fully annotated mammals. (**F**) Comparative analysis of 5’D-12RSS RIC scores and 5’J-12RSS RIC scores for 8 other fully annotated mammals

Further analysis of the effect of the quality of RSSs on the frequency of V or J gene usage revealed that in all the samples from the five mammals, when the V-23RSS RIC score was below -45, its frequency was significantly reduced (while all the J-12RSS RIC scores were higher than -45) (Fig. 7A). The frequency of V and J genes usage correlation with RSSs for each mammal is shown in Supplementary Fig. 3. Among them, the RIC scores of all V genes in mice were significantly positively correlated with the frequency of usage (*P* = 0.0026). However, there was no obvious positive correlation between the frequency of V or J gene usage and RIC scores in humans, rhesus monkeys, bats, or buffaloes. The high or low RIC scores of the distal and proximal V and J genes visibly affected their frequency of use in rearrangement (Supplementary Tables 4 to 8, highlighted in italics). For example, the RIC score of J2-3 (RIC score = -22.56) was the highest in the middle group of the human TRB locus, and its use was prominently higher than that of J2-2, J2-4, J2-5, and J2-6 in the group. The RIC score of V27 (RIC score = -24.85) was highest in the proximal group of the rhesus monkey TRB locus, and the frequency of V27 was significantly higher than that of V28, V29-1, and V30 in the group. The RIC score of V1 (RIC score = -43.05) was lowest in the distal group of the bat TRB locus, and the frequency of V1 was significantly less than that of V3 and V5 (4.77%) in the group.

### Characteristics of V, D, and J RIC scores and V and J genes distances to D gene in mammals

The average V-23RSS RIC scores for human, rhesus monkey, mouse, bat, and buffalo were lower than the average J-12RSS RIC scores (Fig. 7B), whereas the average J-12RSS RIC scores were lower than the average D-12RSS RIC scores (Fig. 7C). The mean distance between the V and D genes in each mammal was significantly greater than the distance between the J and D genes (Fig. 7D). We further analyzed the RIC scores of V, J, and D of the other 8 annotated mammals, which were very consistent with the RIC scores of the 5 sequenced mammals we completed (Fig. 7E, F).

## Discussion

The emergence of VDJC genes on the chromosomes of jawed vertebrates is a pivotal event in the evolution of the “immune explosion” adaptive response. The mechanism and regulatory factors of V(D)J recombination are still being supplemented [6, 10, 11]. V(D)J is not randomly involved in rearrangement, and the physical location of genes on chromosomes [12–14], RSS quality and genetic factors are involved in the regulation of rearrangement [15–18].

In this study, we analyzed the TRB CDR3 repertoires, in which we used HTS and scRNA-seq CDR3 repertoires for humans, mice, and rhesus monkeys. Although there were large differences in the number of CDR3 clonotypes obtained via the two sequencing methods, the results suggested that the effect of distance on the frequency of V and J genes usage was completely consistent in the CDR3 repertoires obtained via the two sequencing methods. Although individual TRBV gene families were found to differ between the HTS and scRNA-seq TRB CDR3 repertoires, this difference may be related to the library construction method and sequencing depth of the two sequencing methods. We utilized the advantages of big data of TCR CDR3 repertoire by HTS and scRNA-seq to analyze the characteristics of V(D)J recombination, and discovered that in the TCRβ CDR3 repertoires of humans, mice, rhesus monkeys, bats, and buffaloes, there was a usage bias in the V and J genes in each sample, and the central and peripheral tissue samples were basically consistent. This suggested that to conduct analysis between mammalian TCRβ CDR3 repertoire and disease, it was necessary to fully consider the “background” characteristics of V(D)J biased recombination in the initial CDR3 repertoire, which determined the individual’s high-clonal response family or defective response family. Among them, T cells with a high frequency of V or J genes usage had more “opportunities” to participate in adaptive responses.

The bias use of the V and J gene subfamily has remained consistent among various mammals. Specifically, in the TRB locus, the distal and proximal V genes “to D” gene showed a significantly higher frequency of use when compared to the middle V genes. According to the rearrangement rules of the mammalian TRB locus, D1 can recombine with J1 and J2, whereas D2 can be rearranged with only J2 (although D2 cannot be directly rearranged with J1 in the 12/23 rule [54], it still has a few special and complex rearrangement patterns). We also observed that the frequency of the proximal and distal J genes “to D” gene demonstrated significant increase compared to the middle J gene. The outcome indicated that the distance of V and J genes “to D” gene in the TRB locus played a dominant role in the efficiency of D-J and V-DJ recombination.

The results of high-frequency use of the proximal V and J groups found in this study aligned with those of the proximal V “to D” of the IGH [12], the proximal J “to D” of the TRB [21], and the proximal J “to V” of the TRG [22] studied in cell models. The mechanism of how distance affects the efficiency of V(D)J recombination has not been described in detail. In 2007, Skok et al. reported that the mouse TRB locus is involved in rearrangement in a loop structure, suggesting that the distal and proximal V genes may have higher accessibility when they recombine with DJ [13]. In 2020, in the scRNA-seq data of the thymus, Park also proposed that the frequencies of the distal and proximal V genes “to D” gene was relatively high and could not be explained by the V-23RSS RIC scores [14]. We observed the dominant involvement of proximal V and J genes “to D” gene in the rearrangement process of five mammals, although the RIC scores were the lowest. The decrease in the average frequency of the proximal V gene in mice was mainly because the proximal V30 RIC score was − 50.42, and the frequency was only 0.33%, resulting in a significant decrease in the average frequency of the proximal V (V29, V30, and V31) genes.

In five mammals, the distal V or J groups were found to be high-frequently used than the middle V or J groups, and in some mammals, they were even more commonly high-frequently used than the proximal V or J groups. The overall quality of the RSSs of the V and J genes were higher than the proximal V and J groups. It suggested that the high-frequency use of distal V and J groups, in addition to the structural recombinant advantage (accessibility) associated with distance, may also be related to the relatively high-quality RSS formed during evolution.

The RIC scores of the middle V and J groups were the highest in the five mammals, but the frequency of use was the lowest, revealing that the D gene and middle V and J groups were structurally inaccessible to each other and played a decisive role in the rearrangement process, “covering the role of the RSS”. The inaccessibility of the middle V in the TRB loop structure recombination model of mice [13] was further confirmed in humans, rhesus monkeys, bats, and buffaloes.

In the annotated TRB locus of mammals (Fig. 7), the RSS quality of the middle V and J groups was relatively higher than that of the proximal group, which may be the result of “positive evolution” to compensate for the disadvantage of recombination in the middle-distance structure. By allowing more V and J genes to participate in rearrangement, the diversity of the CDR3 library can be ensured.

To further analyze the impact of RSS quality on V(D)J rearrangement, our study revealed that V RIC scores below -45 in five mammals significantly decreased the efficiency of V genes participation in rearrangement, which was completely consistent with the results of Gopalakrishnan, S., et al. [16]. These findings indicated that RIC scores below -45 may be used as an independent factor to reduce the participation of the V genes in rearrangement. Regardless of the distance factor, analysis of all V (or J) RIC scores of each mammal was conducted to determine their effect on frequency of use, and it was found that there was no significant correlation between the other four mammals and their V RIC scores with use frequency, except for the mouse V RIC scores, which were significantly positively correlated with frequency of use. However, within the distal-middle-proximal group, the high (or low) RIC scores of most V and J genes were positively correlated with the high (or low) frequency of use. These results revealed that, in addition to the two dominant factors of distance and RSS, a variety of other regulatory molecules exerted positive or negative synergistic effects on the frequency of V and J genes usage. In studies of mice TRB V(D)J rearrangement, factors such as RSS quality, spatial proximity, and chromatin environment have been found to play a role [16]. The regulatory mechanisms of different mammalian RSSs and recombinant proteins, such as RAGs, remain to be further clarified [34].

A comparison of the RIC scores of all the VDJ genes in the 13 annotated mammalian TRB loci revealed that V < J < D. At the same time, the J-D distance was shorter than the V-J distance caused by evolution, suggesting that the regulation of D-J recombination preceding V-DJ recombination in the TRB locus may be related to V-RSS quality and the distance away from the D gene during evolution. The 3’V-23RSS of low quality also prevented V genes from recombining with J-12RSS of relatively low quality directly, thus ensuring that V genes recombine with D-12RSS of relatively high quality in mammalian evolution [34, 55, 56]. Cowell et al. constructed a statistical model (www.itb.cnr.it/rss/RIC) of the RSS quality score to research the impact of RSS quality on VDJ recombination, which was validated and widely applied [14, 16–18, 32, 33]. However, the mechanism for simultaneously analyzing the “combinatorial efficiency of rearrangement” of two RSS qualities in V-D or D-J recombination is still unknown.

The order (sequential) and efficiency (bias) of the V and J genes involved in mammalian TRB rearrangement, in addition to conducting distance and RSS quality research, how to combine multiple factors such as genetics [36, 37], chromatin environment [19], MHC, and other molecules [41] to conduct collaborative research, will be a key direction for analyzing the efficiency of natural VDJ rearrangement under natural conditions and the bias characteristics of the initial TCR CDR3 repertoire. Moreover, the effects of RIC scores on V(D)J rearrangement efficiency need to be investigated in a larger sample of studies and in a larger number of mammalian species to further explore the homogeneity and heterogeneity of the effects of distance and the RSS on V(D)J rearrangement.

## Conclusion

In this study, we used scRNA-seq and HTS to explore the TCRβ CDR3 repertoire of each central and peripheral tissue sample from five mammals (humans, rhesus monkeys, mice, bats, and buffaloes) and found that there was a consistent tendency toward usage bias in the V and J genes. The frequency of distal and proximal V and J genes usage was significantly greater than that of middle V and J genes in the TRB locus. The RSS quality influenced the V and J genes to participate in rearrangement to varying degrees. Although, in theory, a high RIC scores will lead to high usage and a low RIC score will lead to low usage, the RIC score cannot cover the influence of distance on usage, and the influence of distance is stronger than the RIC score. The evolution of RSS and the distance of VDJ genes in the mammalian TRB locus determined the order (sequential) and efficiency (bias) of the V and J genes involved in rearrangement. These results provided a new perspective for studying the rearrangement mechanism, efficiency, and initial CDR3 repertoire characteristics of the mammalian TRB V-D-J.

## Electronic supplementary material

Below is the link to the electronic supplementary material.

Supplementary Material 1

## Data Availability

The raw sequence data of bats, buffaloes, and mice in this experiment were shared in the NCBI database with accession numbers PRJNA877449 (https://www.ncbi.nlm.nih.gov/bioproject/?term=PRJNA877449), PRJNA908273 (https://www.ncbi.nlm.nih.gov/bioproject/?term=PRJNA908273), PRJNA982388 (https://www.ncbi.nlm.nih.gov/bioproject/?term=PRJNA982388), PRJNA906203 (https://www.ncbi.nlm.nih.gov/bioproject/?term=PRJNA906203, and PRJNA982279 (https://www.ncbi.nlm.nih.gov/bioproject/?term=PRJNA982279). In addition to our own data collection, we also utilized publicly available data from shared databases, such as EMBL-EBI and NCBI, for information regarding humans (https://www.ebi.ac.uk/ena/browser/view/PRJEB41936, https://www.ncbi.nlm.nih.gov/geo/query/acc.cgi?acc=GSE168859), rhesus monkeys (https://www.ncbi.nlm.nih.gov/bioproject/PRJNA389234, https://www.ncbi.nlm.nih.gov/bioproject/?term=PRJNA746267), and mice (https://www.ncbi.nlm.nih.gov/geo/query/acc.cgi?acc=GSE168944).

## Declarations

### Declaration of ARRIVE guidelines

We declare that this study is reported in accordance with ARRIVE guidelines (https://arriveguidelines.org).

1. Research design: our research subjects were humans, mice, bats, rhesus monkeys, and buffaloes.
2. Sample size: the human samples were 13, rhesus monkey samples were 6, mouse samples were 18, bat samples were 3, and buffalo sample was 6. Our study did not include a control group, and we compared each group of animals.
3. Randomization: we used a random method to include the study animals.
4. Experimental animal and procedure: (1) In total, 8 human samples were collected: four thymus samples and four peripheral blood samples from infants aged 7, 52, 107, and 156 days. DNA was extracted from PBMCs and thymic cells and sequenced via the ImmunoSEQTM assay and the Illumina platform, and relevant sample information was obtained from the article (10.1016/j.dib.2021.106751). 5 peripheral blood samples were obtained from five healthy donors aged 39, 71, 55, 68, and 41, respectively, PBMCs were isolated using density gradient centrifugation, and single-cell sequencing was performed on the Illumina HiSeq 3000 platform. The basic information of the samples is available in the published article (10.1038/s41467-022-29175-x). (2) Mouse samples: 9 mouse samples were collected from 3 spleen and 3 thymus samples from female BALB/c mice aged 2 months, and 3 thymus samples from Kunming (KM) mice were obtained by our group. Total RNA was extracted from each sample, and the 5’RACE method was used to construct the TCRβ CDR3 repertoires. Sequencing was performed using Illumina NovaSeq 6000 platform. 9 mouse samples from 3 C57BL/6 tumor-bearing mice aged 6–8 weeks were subjected to immunomagnetic bead negative selection to enrich total T cells. Single-cell sequencing was performed on the Illumina HiSeq 4000 platform. The basic sample information is derived from a published article (10.1084/jem.20201329). (3) The source of the rhesus monkey samples was as follows: 1 blood sample was obtained from a 5-year-old healthy female Chinese rhesus monkey. PBMCs were isolated using density gradient centrifugation, and total RNA was then extracted. The TCRβ CDR3 repertoires was constructed using 5’RACE, and sequencing was conducted using the Illumina HiSeq 2000 platform. The basic information of the sample can be found in a previously published article (10.1371/journal.pone.0182733). 4 samples of T cells from the peripheral tissues of rhesus monkeys were collected from PBMCs, splenocytes, and FACS-sorted stimulated nonproliferating and proliferating T cells. TRB sequencing was performed via the Illumina NextSeq 500 platform. The basic information of the sample can be found in a previously published article (10.4049/jimmunol.2100824). (4) Source of the bat samples: 3 bat samples were collected from Rhinolophus affinis in Guizhou, China. Spleen tissue samples were obtained from each bat, and total RNA was extracted. The 5’RACE method was employed to construct the TCRβ CDR3 repertoires, and sequencing was performed using the Illumina HiSeq 1500 platform. (5) The source of the buffalo samples: 6 spleen samples were collected from the buffaloes. The total RNA was reverse transcribed to cDNA. Multiplex PCR was performed to amplify rearranged TCR-β CDR3 sequences. TRB sequencing was performed using the MGISEQ-2000RS platform.
5. Result: all sequencing data were used for the analysis of the TCRβ CDR3 repertoires.
6. Statistical methods: SPSS (version 29.0) was used for the statistical analysis, which was performed using Kruskal-Wallis H Test and Mann Whitney U Test. *P* value < 0.05 was considered statistically significant (**P* value < 0.05, ***P* value < 0.01, ****P* value < 0.001).
