# Supplementary material for: HTS and scRNA-seq revealed that the location and RSS quality of the mammalian TRBV and TRBJ genes impact biased rearrangement
Source: BMC Genomics. 2024 Oct 29;25:1010. doi: 10.1186/s12864-024-10887-x (PMC11520388; doi:10.1186/s12864-024-10887-x)
Supplement: Supplementary file 1 — Supplementary Material 1 [file 12864_2024_10887_MOESM1_ESM.docx]

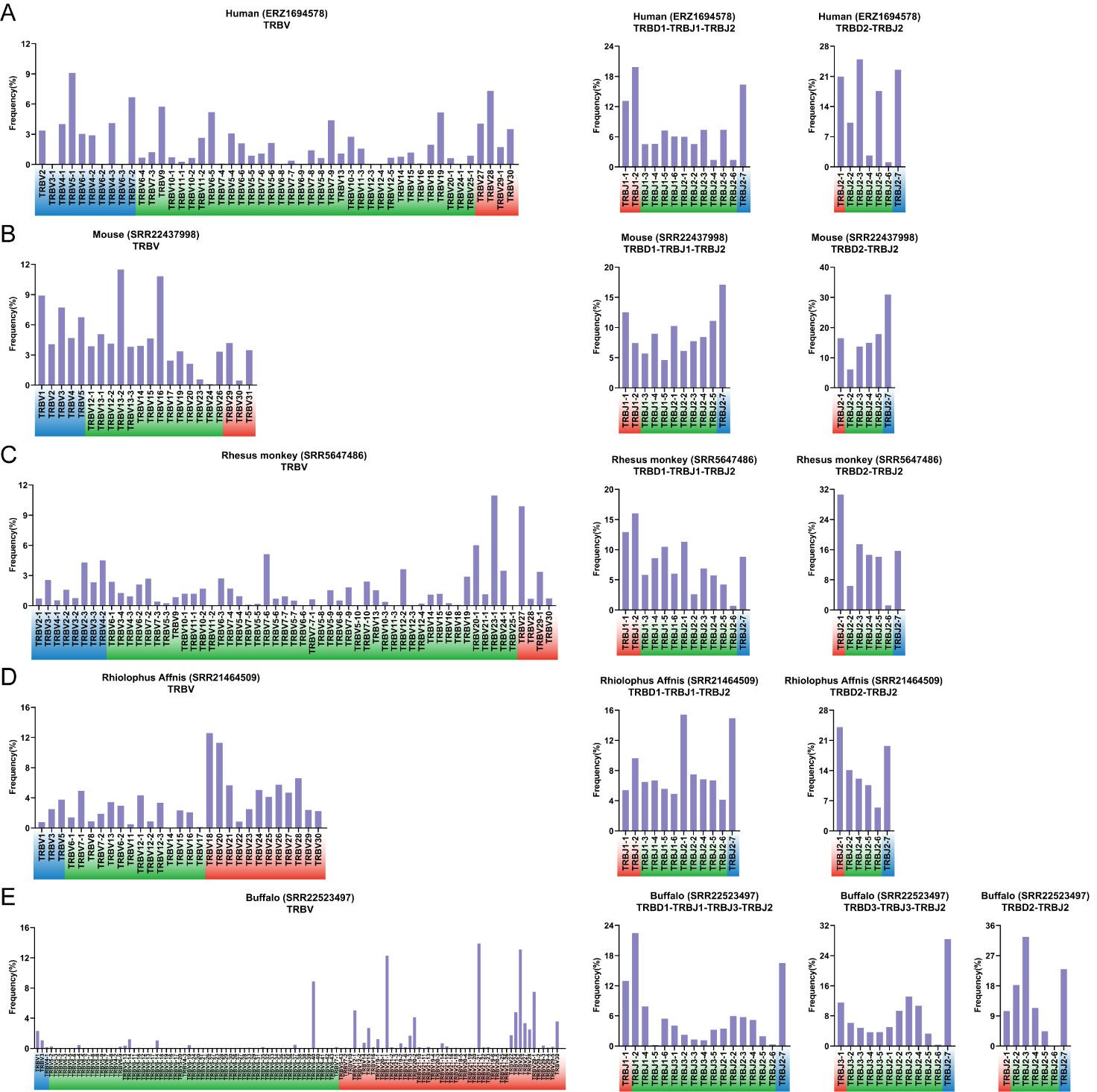


**Sup-Fig. 1** **Example of VDJ usage frequency in TCR CDR3 repertoire of individual samples from Primates (human and rhesus monkey), Rodentia (BALB/c, C57BL/6, and KM mice), Artiodactyla (buffalo), and Chiroptera (*Rhinolophus affinis*) (HTS). (A)** Human (ERZ1694578). **(B)** Mouse (SRR22437998). **(C)** Rhesus monkey (SRR5647486). **(D)** *Rhinolophus affinis* (SRR21464509). **(E)** Buffalo (SRR22523497).


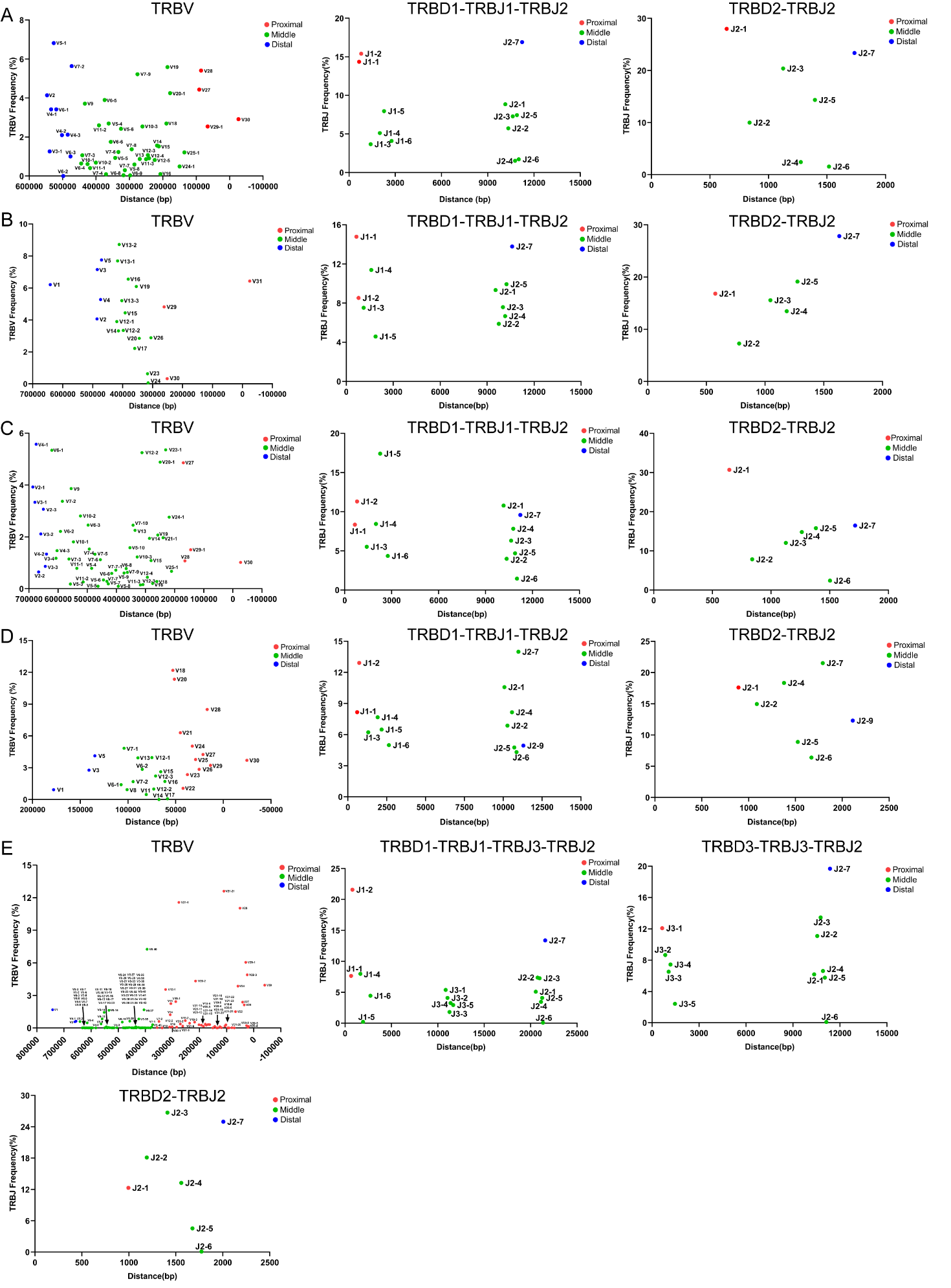


**Sup-Fig. 2** **Correlation between the use of V and J genes and specific locations in human, mouse, monkey, bat and buffalo. (A)** Total human samples (n = 13). **(B)** Total mouse samples (n = 18). **(C)** Total monkey samples (n = 6). **(D)** Total bat samples (n = 3). **(E)** Total buffalo samples (n = 6).


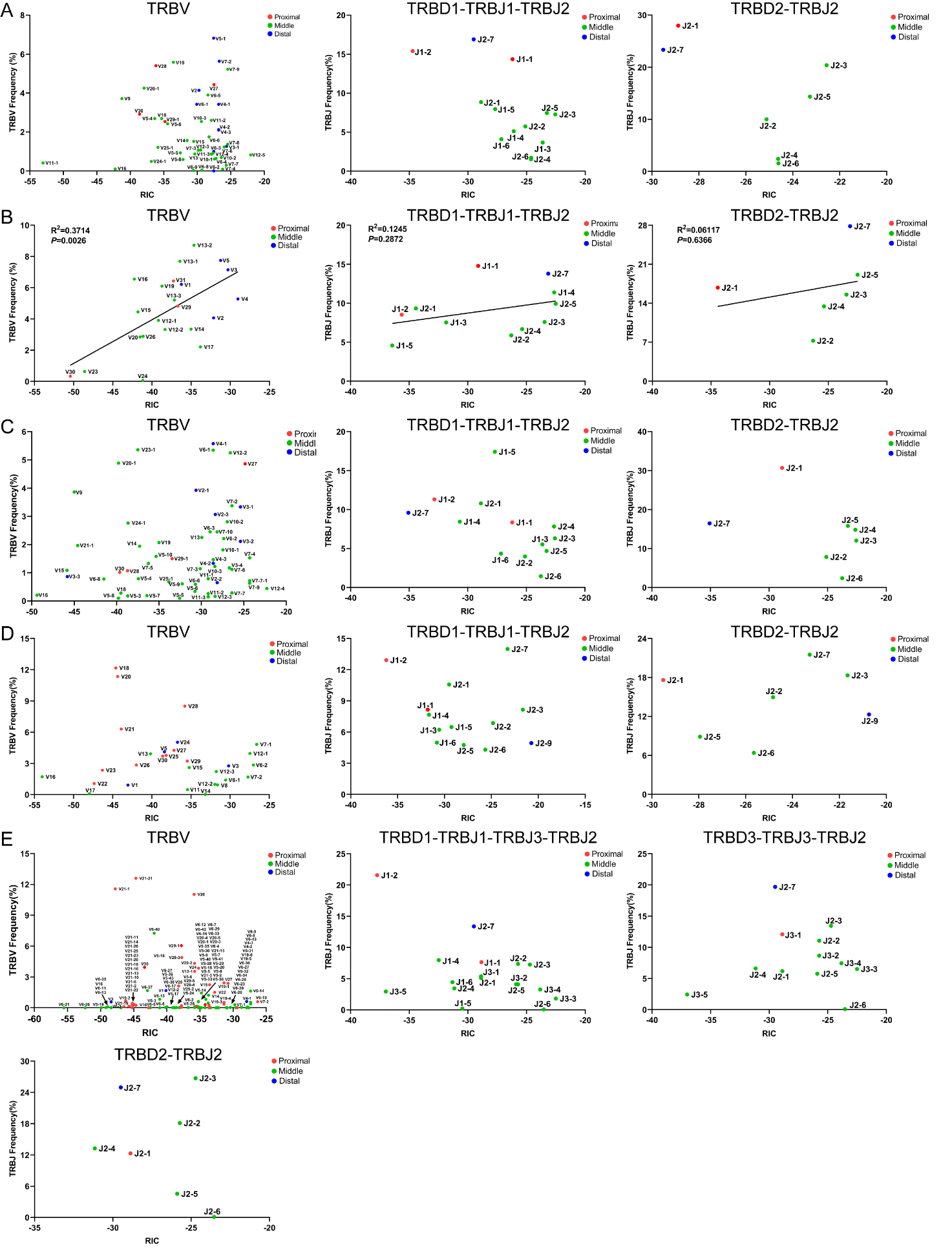


**Sup-Fig. 3 Correlation between V and J usage frequency and RIC scores** **in human, mouse, monkey, bat and buffalo. (A)** Total human samples (n = 13). **(B)** Total mouse samples (n = 18). **(C)** Total monkey samples (n = 6). **(D)** Total bat samples (n = 3). **(E)** Total buffalo samples (n = 6).

**Supplementary Table 1. Basic information of human samples and analysis of unique TCRβ CDR3 sequences.**

| **Accession Number** | **Species** | **Tissue** | **Starting material** | **Library Preparation approach** | **Sequencing** | **Analysis Sequence Clonetype** |
| --- | --- | --- | --- | --- | --- | --- |
| ERZ1694549 | *Homo sapiens* | Thymus | DNA | multiplex PCR | HTS | 96616 |
| ERZ1694551 | *Homo sapiens* | Thymus | DNA | multiplex PCR | HTS | 81013 |
| ERZ1694560 | *Homo sapiens* | Thymus | DNA | multiplex PCR | HTS | 61308 |
| ERZ1694569 | *Homo sapiens* | Thymus | DNA | multiplex PCR | HTS | 55729 |
| ERZ1694578 | *Homo sapiens* | Blood | DNA | multiplex PCR | HTS | 33817 |
| ERZ1694579 | *Homo sapiens* | Blood | DNA | multiplex PCR | HTS | 31686 |
| ERZ1694580 | *Homo sapiens* | Blood | DNA | multiplex PCR | HTS | 46336 |
| ERZ1694581 | *Homo sapiens* | Blood | DNA | multiplex PCR | HTS | 35756 |
| GSM5171626 | *Homo sapiens* | Blood | RNA | Nested PCR | ScRNA-seq | 12394 |
| GSM5171627 | *Homo sapiens* | Blood | RNA | Nested PCR | ScRNA-seq | 13473 |
| GSM5171634 | *Homo sapiens* | Blood | RNA | Nested PCR | ScRNA-seq | 8059 |
| GSM5171635 | *Homo sapiens* | Blood | RNA | Nested PCR | ScRNA-seq | 7223 |
| GSM5171642 | *Homo sapiens* | Blood | RNA | Nested PCR | ScRNA-seq | 50743 |

**Supplementary Table 2. Basic information of mouse samples and analysis of unique TCRβ CDR3 sequences.**

| **Accession**  **Number** | **Species** | **Tissue** | **Starting material** | **Library Preparation approach** | **Sequencing** | **Analysis Sequence Clonetype** |
| --- | --- | --- | --- | --- | --- | --- |
| SRR22438002 | *Mus musculus* | Thymus | RNA | 5’RACE | HTS | 795330 |
| SRR22438001 | *Mus musculus* | Thymus | RNA | 5’RACE | HTS | 538438 |
| SRR22438000 | *Mus musculus* | Thymus | RNA | 5’RACE | HTS | 465501 |
| SRR24908413 | *Mus musculus* | Thymus | RNA | 5’RACE | HTS | 740204 |
| SRR24908412 | *Mus musculus* | Thymus | RNA | 5’RACE | HTS | 846904 |
| SRR24908411 | *Mus musculus* | Thymus | RNA | 5’RACE | HTS | 678187 |
| SRR22437999 | *Mus musculus* | Spleen | RNA | 5’RACE | HTS | 416140 |
| SRR22437998 | *Mus musculus* | Spleen | RNA | 5’RACE | HTS | 582692 |
| SRR22437997 | *Mus musculus* | Spleen | RNA | 5’RACE | HTS | 766508 |
| GSM5172690 | *Mus musculus* | Lymph node | RNA | Nested PCR | scRNA-seq | 7226 |
| GSM5172691 | *Mus musculus* | Lymph node | RNA | Nested PCR | scRNA-seq | 7803 |
| GSM5172698 | *Mus musculus* | Lymph node | RNA | Nested PCR | scRNA-seq | 3088 |
| GSM5172688 | *Mus musculus* | Spleen | RNA | Nested PCR | scRNA-seq | 10158 |
| GSM5172689 | *Mus musculus* | Spleen | RNA | Nested PCR | scRNA-seq | 6669 |
| GSM5172696 | *Mus musculus* | Spleen | RNA | Nested PCR | scRNA-seq | 5367 |
| GSM5172686 | *Mus musculus* | Blood | RNA | Nested PCR | scRNA-seq | 5155 |
| GSM5172687 | *Mus musculus* | Blood | RNA | Nested PCR | scRNA-seq | 5947 |
| GSM5172694 | *Mus musculus* | Blood | RNA | Nested PCR | scRNA-seq | 2777 |

**Supplementary Table 3. Basic information of bat, rhesus monkey, and buffalo samples and analysis of unique TCRβ CDR3 sequences.**

| **Accession Number** | **Species** | **Tissue** | **Starting material** | **Library Preparation approach** | **Sequencing** | **Analysis Sequence Clonetype** |
| --- | --- | --- | --- | --- | --- | --- |
| SRR21464510 | *Rhiolophus Affnis* | Spleen | DNA | multiplex PCR | HTS | 1770 |
| SRR21464509 | *Rhiolophus Affnis* | Spleen | DNA | multiplex PCR | HTS | 11839 |
| SRR21464508 | *Rhiolophus Affnis* | Spleen | DNA | multiplex PCR | HTS | 23874 |
| SRR5647486 | *Macaca mulatta* | Blood | RNA | 5’RACE | HTS | 140355 |
| SRR15249798 | *Macaca mulatta* | Spleen | RNA | Nested PCR | scRNA-seq | 5222 |
| SRR15249806 | *Macaca mulatta* | Blood | RNA | Nested PCR | scRNA-seq | 5496 |
| SRR15249810 | *Macaca mulatta* | Blood | RNA | Nested PCR | scRNA-seq | 6486 |
| SRR15249812 | *Macaca mulatta* | Blood | RNA | Nested PCR | scRNA-seq | 21819 |
| SRR15249814 | *Macaca mulatta* | Blood | RNA | Nested PCR | scRNA-seq | 29906 |
| SRR24889447 | *Bubalus bubalis* | Spleen | DNA | multiplex PCR | HTS | 29641 |
| SRR24889446 | *Bubalus bubalis* | Spleen | DNA | multiplex PCR | HTS | 18623 |
| SRR24889445 | *Bubalus bubalis* | Spleen | DNA | multiplex PCR | HTS | 17578 |
| SRR24889444 | *Bubalus bubalis* | Spleen | DNA | multiplex PCR | HTS | 34392 |
| SRR24889443 | *Bubalus bubalis* | Spleen | DNA | multiplex PCR | HTS | 13814 |
| SRR22523497 | *Bubalus bubalis* | Spleen | DNA | multiplex PCR | HTS | 32118 |

**Supplementary Table 4. Names of V, D, and J, physical distance (bp) of the V or J to D gene and grouping, RSS RIC scores, and frequency of use in human TRB locus.**

Note. **D1-12RSS (RIC score = -20.18), D1-23RSS (RIC score = -36.20), D2-12RSS (RIC score = -24.89), and D2-23RSS (RIC score = -44.76).**

| **Gene** | **Distance**  **(bp)** | **RIC** | **Frequency** | **Gene** | **Distance**  **(bp)** | **RIC** | **Frequency** |
| --- | --- | --- | --- | --- | --- | --- | --- |
| V2 | 546281 | -29.75 | 4.14% | V12-4 | 241180 | -27.65 | 0.91% |
| V3-1 | 538658 | -25.62 | 1.26% | V12-5 | 223989 | -22.04 | 0.82% |
| V4-1 | 534040 | -26.82 | 3.42% | V14 | 217060 | -31.53 | 1.56% |
| V5-1 | 526176 | -27.57 | 6.82% | V15 | 211949 | -30.54 | 1.52% |
| V6-1 | 518932 | -30.08 | 3.42% | ***V16*** | ***206955*** | ***-42.26*** | ***0.10%*** |
| V4-2 | 501736 | -26.82 | 2.10% | V18 | 189103 | -35.33 | 2.69% |
| V6-2 | 497937 | -27.55 | 0.00% | V19 | 185986 | -33.57 | 5.59% |
| V4-3 | 484476 | -26.82 | 2.12% | V20-1 | 178119 | -37.95 | 4.25% |
| V6-3 | 476256 | -27.55 | 1.00% | V24-1 | 149751 | -36.87 | 0.49% |
| V7-2 | 472559 | -26.73 | 5.64% | V25-1 | 135385 | -35.88 | 1.21% |
| V6-4 | 444311 | -27.20 | 0.64% | V27 | 90757 | -27.50 | 4.43% |
| V7-3 | 440718 | -29.86 | 1.06% | V28 | 85459 | -36.18 | 5.41% |
| V9 | 433146 | -41.26 | 3.71% | V29-1 | 65708 | -34.82 | 2.54% |
| V10-1 | 425180 | -27.46 | 0.61% | V30 | 26373 | -38.62 | 2.91% |
| ***V11-1*** | ***417423*** | ***-53.05*** | ***0.41%*** | D1-J1-1 | 654 | -26.2 | 14.35% |
| V10-2 | 400114 | -26.41 | 0.69% | D1-J1-2 | 791 | -34.72 | 15.40% |
| V11-2 | 391165 | -27.90 | 2.60% | D1-J1-3 | 1404 | -23.63 | 3.67% |
| V6-5 | 374111 | -28.40 | 3.90% | D1-J1-4 | 1999 | -26.1 | 5.10% |
| V7-4 | 369927 | -26.21 | 0.09% | D1-J1-5 | 2272 | -27.68 | 7.95% |
| V5-4 | 361977 | -36.36 | 2.69% | D1-J1-6 | 2762 | -27.16 | 4.09% |
| V6-6 | 355539 | -28.24 | 1.75% | D1-J2-1 | 10139 | -28.88 | 8.84% |
| V5-5 | 342532 | -32.54 | 0.93% | D1-J2-2 | 10334 | -25.12 | 5.73% |
| V7-6 | 332881 | -25.98 | 1.23% | D1-J2-3 | 10621 | -22.56 | 7.27% |
| V5-6 | 325019 | -34.43 | 2.42% | D1-J2-4 | 10772 | -24.63 | 1.52% |
| V6-8 | 317744 | -29.33 | 0.04% | D1-J2-5 | 10893 | -23.27 | 7.45% |
| V7-7 | 313429 | -25.67 | 0.30% | D1-J2-6 | 11013 | -24.61 | 1.71% |
| V6-9 | 297730 | -30.57 | 0.03% | D1-J2-7 | 11230 | -29.52 | 16.90% |
| V7-8 | 293064 | -25.47 | 1.37% | D2-J2-1 | 643 | -28.88 | 28.00% |
| V5-8 | 284865 | -32.14 | 0.59% | D2-J2-2 | 838 | -25.12 | 10.00% |
| V7-9 | 275658 | -25.45 | 5.22% | ***D2-J2-3*** | ***1125*** | ***-22.56*** | ***20.38%*** |
| V13 | 269128 | -30.38 | 0.87% | D2-J2-4 | 1276 | -24.63 | 2.39% |
| V10-3 | 260750 | -29.39 | 2.54% | ***D2-J2-5*** | ***1397*** | ***-23.27*** | ***14.35%*** |
| V11-3 | 250114 | -27.90 | 0.86% | D2-J2-6 | 1517 | -24.61 | 1.53% |
| V12-3 | 244503 | -29.50 | 1.07% | D2-J2-7 | 1734 | -29.52 | 23.36% |

**Supplementary Table 5. Names of V, D, and J, physical distance (bp) of the V or J to D gene and grouping, RSS RIC scores, and frequency of use in mouse TRB locus.**

Note. **D1-12RSS (RIC score = -23.05), D1-23RSS (RIC score = -35.73), D2-12RSS (RIC score = -22.34), and D2-23RSS (RIC score = -40.68).**

| **Gene** | **Distance**  **(bp)** | **RIC** | **Frequency** | **Gene** | **Distance**  **(bp)** | **RIC** | **Frequency** |
| --- | --- | --- | --- | --- | --- | --- | --- |
| V1 | 641315 | -36.24 | 6.21% | V30 | 251210 | -50.42 | 0.33% |
| V2 | 485205 | -32.14 | 4.07% | V31 | 24493 | -37.26 | 6.43% |
| V3 | 484376 | -30.29 | 7.15% | D1-J1-1 | 650 | -29.11 | 14.78% |
| V4 | 473314 | -29.03 | 5.28% | D1-J1-2 | 787 | -35.64 | 8.52% |
| V5 | 470397 | -31.23 | 7.75% | D1-J1-3 | 1109 | -31.84 | 7.53% |
| V12-1 | 419133 | -39.17 | 3.91% | ***D1-J1-4*** | ***1597*** | ***-22.63*** | ***11.39%*** |
| V13-1 | 416732 | -36.43 | 7.69% | D1-J1-5 | 1870 | -36.45 | 4.58% |
| V12-2 | 413836 | -38.33 | 3.32% | D1-J2-1 | 9540 | -34.42 | 9.33% |
| V13-2 | 411368 | -34.64 | 8.72% | D1-J2-2 | 9743 | -26.29 | 5.88% |
| V13-3 | 402615 | -37.13 | 5.21% | D1-J2-3 | 10009 | -23.45 | 7.59% |
| V14 | 397584 | -35.01 | 3.35% | D1-J2-4 | 10148 | -25.36 | 6.67% |
| V15 | 391542 | -41.79 | 4.45% | D1-J2-5 | 10239 | -22.49 | 9.93% |
| V16 | 380970 | -42.25 | 6.55% | D1-J2-7 | 10596 | -23.13 | 13.79% |
| V17 | 359644 | -33.80 | 2.21% | D2-J2-1 | 576 | -34.42 | 16.81% |
| V19 | 354152 | -38.72 | 6.10% | D2-J2-2 | 779 | -26.29 | 7.26% |
| V20 | 344223 | -41.51 | 2.85% | D2-J2-3 | 1045 | -23.45 | 15.56% |
| ***V23*** | ***316674*** | ***-48.60*** | ***0.63%*** | D2-J2-4 | 1184 | -25.36 | 13.44% |
| V24 | 314643 | -41.17 | 0.06% | ***D2-J2-5*** | ***1275*** | ***-22.49*** | ***19.11%*** |
| V26 | 305287 | -41.12 | 2.89% | D2-J2-7 | 1632 | -23.13 | 27.83% |
| V29 | 261319 | -36.69 | 4.82% |  |  |  |  |

**Supplementary Table 6. Names of V, D, and J, physical distance (bp) of the V or J to D gene and grouping, RSS RIC scores, and frequency of use in rhesus monkey TRB locus.**

Note. **D1-12RSS (RIC score = -21.13), D1-23RSS (RIC score = -36.90), D2-12RSS (RIC score = -24.89), and D2-23RSS (RIC score = -41.96).**

| **Gene** | **Distance**  **(bp)** | **RIC** | **Frequency** | **Gene** | **Distance**  **(bp)** | **RIC** | **Frequency** |
| --- | --- | --- | --- | --- | --- | --- | --- |
| V2-1 | 686521 | -30.64 | 3.92% | V11-3 | 317356 | -29.20 | 0.15% |
| V3-1 | 679552 | -25.40 | 3.33% | V12-2 | 311720 | -26.59 | 5.25% |
| V4-1 | 674914 | -28.61 | 5.57% | V12-3 | 308454 | -28.38 | 0.16% |
| V2-2 | 666690 | -28.14 | 0.65% | V12-4 | 293116 | -22.28 | 0.44% |
| V3-2 | 659093 | -25.40 | 2.11% | V14 | 286137 | -37.28 | 1.94% |
| V2-3 | 650580 | -28.36 | 3.07% | ***V15*** | ***280654*** | ***-45.84*** | ***1.08%*** |
| ***V3-3*** | ***644077*** | ***-45.81*** | ***0.86%*** | ***V16*** | ***275367*** | ***-49.39*** | ***0.20%*** |
| V4-2 | 639712 | -28.61 | 1.33% | V18 | 260736 | -39.49 | 0.28% |
| V6-1 | 621477 | -28.61 | 5.35% | V19 | 257499 | -35.00 | 2.07% |
| V3-4 | 606825 | -26.71 | 1.17% | V20-1 | 249544 | -39.77 | 4.89% |
| V4-3 | 603392 | -28.61 | 1.47% | V21-1 | 239438 | -44.59 | 1.97% |
| V6-2 | 591643 | -27.36 | 2.21% | V23-1 | 230474 | -37.50 | 5.36% |
| V7-2 | 585254 | -26.40 | 3.37% | V24-1 | 218333 | -38.65 | 2.76% |
| V7-3 | 562714 | -30.14 | 1.14% | V25-1 | 210501 | -33.83 | 0.67% |
| V5-3 | 557672 | -38.69 | 0.18% | ***V27*** | ***169925*** | ***-24.85*** | ***4.86%*** |
| V9 | 554989 | -44.99 | 3.86% | V28 | 164585 | -38.70 | 1.07% |
| V10-1 | 546984 | -27.46 | 1.81% | V29-1 | 144741 | -33.46 | 1.50% |
| V11-1 | 535545 | -29.20 | 0.79% | V30 | 26604 | -39.64 | 1.02% |
| V10-2 | 522972 | -26.95 | 2.81% | D1-J1-1 | 656 | -26.2 | 7.44% |
| V11-2 | 513814 | -29.20 | 0.26% | D1-J1-2 | 790 | -32.84 | 10.37% |
| V6-3 | 496705 | -28.97 | 2.45% | D1-J1-3 | 1409 | -23.63 | 5.47% |
| V7-4 | 492478 | -24.32 | 1.53% | D1-J1-4 | 1997 | -30.68 | 8.41% |
| V5-4 | 484625 | -37.43 | 0.79% | D1-J1-5 | 2268 | -27.68 | 18.81% |
| V7-5 | 473176 | -36.25 | 1.33% | D1-J1-6 | 2757 | -27.16 | 4.01% |
| V5-5 | 462708 | -32.56 | 0.10% | D1-J2-1 | 10152 | -28.88 | 10.69% |
| V7-6 | 454759 | -26.40 | 1.12% | D1-J2-2 | 10347 | -25.12 | 4.25% |
| V5-6 | 444118 | -30.76 | 0.34% | D1-J2-3 | 10634 | -22.56 | 6.19% |
| V7-7 | 432560 | -26.40 | 0.28% | D1-J2-4 | 10770 | -22.64 | 8.24% |
| V5-7 | 427495 | -36.43 | 0.19% | D1-J2-5 | 10890 | -23.27 | 4.77% |
| V6-6 | 415759 | -30.71 | 0.59% | D1-J2-6 | 11010 | -23.76 | 1.60% |
| V7-7-1 | 401627 | -24.32 | 0.72% | D1-J2-7 | 11224 | -35.07 | 9.74% |
| V5-8 | 393170 | -39.78 | 0.10% | D2-J2-1 | 644 | -28.88 | 30.72% |
| V5-9 | 373345 | -32.26 | 0.60% | D2-J2-2 | 839 | -25.12 | 8.12% |
| V6-8 | 366177 | -41.51 | 0.78% | D2-J2-3 | 1126 | -22.56 | 10.96% |
| V7-9 | 361865 | -24.32 | 0.63% | D2-J2-4 | 1262 | -22.64 | 14.84% |
| V5-10 | 353016 | -35.34 | 1.58% | D2-J2-5 | 1382 | -23.27 | 16.15% |
| V7-10 | 342290 | -28.15 | 2.45% | D2-J2-6 | 1502 | -23.76 | 2.60% |
| V13 | 335522 | -29.96 | 2.25% | D2-J2-7 | 1716 | -35.07 | 16.61% |
| V10-3 | 328313 | -28.41 | 1.23% |  |  |  |  |

**Supplementary Table 7. Names of V, D, and J, physical distance (bp) of the V or J to D gene and grouping, RSS RIC scores, and frequency of use in bat TRB locus.**

Note. **(1) In the annotated *Rhinolophus ferrumequinum* TRB CDR3 repertoire, TRBJ8 and TRBJ9 (different from other mammals) exist after TRBJ2-7, and there was matching involvement in rearrangement of TRBJ9 in sequenced *Rhinolophus affinis*. (2) D1-12RSS (RIC score = -20.85), D1-23RSS (RIC score = -36.90), D2-12RSS (RIC score = -24.79), and D2-23RSS (RIC score = -46.71).**

| **Gene** | **Distance**  **(bp)** | **RIC** | **Frequency** | **Gene** | **Distance**  **(bp)** | **RIC** | **Frequency** |
| --- | --- | --- | --- | --- | --- | --- | --- |
| ***V1*** | ***177912*** | ***-43.05*** | ***0.92%*** | V28 | 16848 | -35.78 | 8.50% |
| V3 | 140815 | -30.19 | 2.77% | V29 | 13369 | -35.47 | 3.22% |
| V5 | 134620 | -38.39 | 4.11% | V30 | 24818 | -38.62 | 3.70% |
| V6-1 | 107083 | -30.56 | 1.40% | D1-J1-1 | 599 | -31.79 | 8.16% |
| V7-1 | 104006 | -26.61 | 4.83% | D1-J1-2 | 734 | -36.21 | 12.91% |
| V8 | 100856 | -31.62 | 0.93% | D1-J1-3 | 1318 | -30.56 | 6.22% |
| V7-2 | 94529 | -27.70 | 1.69% | D1-J1-4 | 1917 | -31.65 | 7.67% |
| V13 | 89430 | -40.17 | 3.92% | D1-J1-5 | 2173 | -29.25 | 6.48% |
| V6-2 | 84981 | -27.02 | 2.85% | D1-J1-6 | 2646 | -30.81 | 4.98% |
| V11 | 80765 | -35.42 | 0.48% | D1-J2-1 | 10073 | -29.51 | 10.57% |
| V12-1 | 74825 | -27.46 | 3.95% | D1-J2-2 | 10269 | -24.83 | 6.87% |
| V12-2 | 72877 | -31.94 | 0.99% | D1-J2-4 | 10559 | -21.65 | 8.15% |
| V12-3 | 70958 | -31.76 | 2.22% | D1-J2-5 | 10706 | -27.95 | 4.75% |
| V14 | 67419 | -33.18 | 0.01% | D1-J2-6 | 10852 | -25.65 | 4.31% |
| V15 | 65536 | -35.22 | 2.61% | D1-J2-7 | 10973 | -23.27 | 13.99% |
| ***V16*** | ***61268*** | ***-54.01*** | ***1.71%*** | D1-J2-8 | 11075 | -23.99 | 0% |
| ***V17*** | ***58511*** | ***-47.98*** | ***0.08%*** | D1-J2-9 | 11293 | -20.74 | 4.93% |
| V18 | 52883 | -44.59 | 12.18% | D2-J2-1 | 892 | -29.51 | 17.61% |
| V20 | 51258 | -44.37 | 11.35% | D2-J2-2 | 1088 | -24.83 | 14.97% |
| V21 | 45019 | -43.91 | 6.30% | D2-J2-4 | 1378 | -21.65 | 18.33% |
| ***V22*** | ***42151*** | ***-47.37*** | ***1.06%*** | D2-J2-5 | 1525 | -27.95 | 8.88% |
| ***V23*** | ***37575*** | ***-46.31*** | ***2.35%*** | D2-J2-6 | 1671 | -25.65 | 6.38% |
| V24 | 32466 | -36.72 | 5.04% | D2-J2-7 | 1792 | -23.27 | 21.51% |
| V25 | 29027 | -38.16 | 3.77% | D1-J2-8 | 1894 | -23.99 | 0% |
| V26 | 25303 | -41.97 | 2.85% | D1-J2-9 | 2112 | -20.74 | 12.31% |
| V27 | 21265 | -37.16 | 4.25% |  |  |  |  |

**Supplementary Table 8. Names of V, D, and J, physical distance (bp) of the V or J to D gene and grouping, RSS RIC scores, and frequency of use in buffalo TRB locus.**

Note. **D1-12RSS (RIC score = -24.07), D1-23RSS (RIC score = -42.05), D3-12RSS (RIC score = -22.17), D3-23RSS (RIC score = -49.23), D2-12RSS (RIC score = -22.56), and D2-23RSS (RIC score = -49.23).**

| **Gene** | **Distance**  **(bp)** | **RIC** | **Frequency** | **Gene** | **Distance**  **(bp)** | **RIC** | **Frequency** |
| --- | --- | --- | --- | --- | --- | --- | --- |
| V1 | 741658 | -40.13 | 1.68% | V20-1 | 283938 | -33.62 | 0.07% |
| V3 | 659549 | -48.41 | 0.57% | ***V21-1*** | ***276096*** | ***-47.78*** | ***11.56%*** |
| V4-1 | 655254 | -27.94 | 0.61% | ***V21-2*** | ***270592*** | ***-45.22*** | ***0.36%*** |
| V5-2 | 640642 | -35.24 | 0.58% | V21-3 | 261549 | -35.51 | 0.16% |
| V5-3 | 632005 | -41.08 | 0.00% | ***V19-2*** | ***252235*** | ***-46.21*** | ***0.67%*** |
| V6-2 | 626795 | -36.76 | 0.02% | ***V21-6*** | ***236169*** | ***-45.22*** | ***0.44%*** |
| ***V6-3*** | ***622891*** | ***-46.93*** | ***0.00%*** | V19-3 | 217913 | -31.37 | 0.54% |
| V5-5 | 620677 | -35.24 | 0.00% | V20-2 | 214301 | -35.82 | 4.33% |
| V5-6 | 616049 | -36.76 | 0.01% | ***V21-10*** | ***201753*** | ***-45.22*** | ***0.29%*** |
| V6-4 | 614238 | -33.75 | 0.47% | V21-11 | 192680 | -44.67 | 0.25% |
| V6-5 | 610676 | -42.50 | 0.00% | ***V21-13*** | ***185244*** | ***-45.22*** | ***0.14%*** |
| V5-7 | 607932 | -34.78 | 0.00% | V19-4 | 178305 | -31.37 | 0.34% |
| V6-6 | 606289 | -41.05 | 0.00% | V20-3 | 174734 | -33.62 | 0.26% |
| V5-8 | 604073 | -35.24 | 0.00% | V21-14 | 167846 | -44.93 | 0.31% |
| V5-9 | 599651 | -34.91 | 0.01% | V21-15 | 162282 | -34.21 | 0.30% |
| V6-7 | 597856 | -32.19 | 0.00% | ***V21-16*** | ***150496*** | ***-45.22*** | ***0.00%*** |
| V6-8 | 594292 | -27.62 | 0.00% | ***V21-18*** | ***143642*** | ***-45.22*** | ***0.14%*** |
| V4-2 | 582330 | -27.94 | 0.00% | V19-5 | 136230 | -29.37 | 0.00% |
| V6-9 | 562813 | -27.36 | 0.37% | V20-4 | 132625 | -33.62 | 0.00% |
| V5-13 | 559034 | -41.08 | 0.83% | ***V21-19*** | ***124450*** | ***-46.48*** | ***0.12%*** |
| V5-14 | 551555 | -34.78 | 1.43% | ***V21-20*** | ***118946*** | ***-45.22*** | ***0.02%*** |
| ***V6-11*** | ***549916*** | ***-48.39*** | ***0.01%*** | V21-21 | 109904 | -44.67 | 12.60% |
| V5-16 | 543311 | -35.24 | 0.10% | ***V21-22*** | ***105884*** | ***-45.72*** | ***0.16%*** |
| V6-12 | 541498 | -32.22 | 0.02% | ***V21-23*** | ***102474*** | ***-45.22*** | ***0.28%*** |
| ***V6-13*** | ***537934*** | ***-48.38*** | ***0.02%*** | V19-6 | 95059 | -29.37 | 0.00% |
| V5-17 | 535301 | -40.21 | 0.04% | V20-5 | 91545 | -33.62 | 0.01% |
| V6-14 | 533661 | -27.36 | 1.63% | ***V21-25*** | ***80325*** | ***-45.22*** | ***0.02%*** |
| V5-18 | 530532 | -41.08 | 0.02% | ***V21-26*** | ***71529*** | ***-45.22*** | ***0.00%*** |
| ***V5-19*** | ***523044*** | ***-49.92*** | ***0.00%*** | V22 | 66995 | -32.78 | 1.51% |
| V6-16 | 521405 | -33.40 | 0.00% | V24 | 58015 | -35.21 | 3.85% |
| V6-17 | 517838 | -39.13 | 0.02% | V26 | 50535 | -35.89 | 11.03% |
| V5-20 | 515441 | -35.24 | 0.00% | V27 | 40741 | -30.76 | 2.38% |
| V4-3 | 504371 | -27.94 | 0.02% | V28 | 34919 | -38.21 | 2.13% |
| V6-19 | 484852 | -27.36 | 0.54% | V29-1 | 29183 | -37.81 | 6.05% |
| V5-24 | 481091 | -38.12 | 0.02% | V29-2 | 26183 | -37.81 | 0.21% |
| V6-20 | 475882 | -31.03 | 0.01% | V29-3 | 23697 | -37.74 | 4.90% |
| ***V6-21*** | ***471974*** | ***-55.53*** | ***0.00%*** | V29-4 | 21221 | -37.81 | 0.04% |
| V5-26 | 469762 | -36.76 | 0.03% | V29-5 | 18755 | -37.81 | 0.14% |

**Refer to Supplementary Table 8**

| **Gene** | **Distance**  **(bp)** | **RIC** | **Frequency** | ***Gene*** | **Distance**  **(bp)** | **RIC** | **Frequency** |
| --- | --- | --- | --- | --- | --- | --- | --- |
| V5-27 | 464788 | -38.54 | 0.02% | V30 | 40822 | -43.36 | 3.95% |
| V6-23 | 459405 | -30.41 | 0.03% | D1-J1-1 | 652 | -28.85 | 7.64% |
| V5-28 | 457117 | -35.24 | 0.62% | D1-J1-2 | 786 | -37.74 | 21.58% |
| V5-29 | 451180 | -35.24 | 0.01% | D1-J1-4 | 1659 | -32.48 | 7.97% |
| V5-30 | 447207 | -34.23 | 0.02% | D1-J1-5 | 1935 | -30.47 | 0.20% |
| ***V6-26*** | ***444709*** | ***-52.32*** | ***0.00%*** | D1-J1-6 | 2732 | -31.32 | 4.44% |
| V6-27 | 440711 | -29.60 | 0.01% | D1-J3-1 | 10838 | -28.88 | 5.37% |
| V6-28 | 437034 | -30.41 | 0.01% | D1-J3-2 | 11034 | -25.73 | 4.09% |
| V5-33 | 434828 | -35.81 | 0.11% | D1-J3-3 | 11252 | -22.50 | 1.80% |
| V6-29 | 433010 | -32.41 | 0.01% | D1-J3-4 | 11369 | -23.84 | 3.26% |
| V6-31 | 426068 | -28.72 | 0.04% | D1-J3-5 | 11656 | -37.00 | 2.94% |
| V5-35 | 423831 | -34.11 | 0.83% | D1-J2-1 | 20550 | -28.88 | 5.09% |
| V6-32 | 421984 | -29.62 | 0.03% | D1-J2-2 | 20746 | -25.73 | 7.37% |
| V6-33 | 418684 | -32.60 | 0.00% | D1-J2-3 | 20966 | -24.72 | 7.26% |
| V6-34 | 415545 | -30.41 | 0.00% | D1-J2-4 | 21113 | -31.16 | 3.45% |
| V5-36 | 413319 | -38.70 | 0.01% | D1-J2-5 | 21232 | -25.89 | 4.09% |
| V6-35 | 411490 | -49.18 | 0.03% | D1-J2-6 | 21328 | -23.53 | 0.07% |
| V6-36 | 408196 | -29.60 | 0.01% | D1-J2-7 | 21560 | -29.49 | 13.37% |
| V6-37 | 404544 | -42.93 | 1.67% | D3-J3-1 | 641 | -28.88 | 12.09% |
| V5-38 | 402322 | -35.81 | 0.02% | D3-J3-2 | 837 | -25.73 | 8.66% |
| V6-38 | 400206 | -38.86 | 0.00% | D3-J3-3 | 1055 | -22.50 | 6.52% |
| V6-39 | 396905 | -30.90 | 0.00% | D3-J3-4 | 1172 | -23.84 | 7.44% |
| V6-40 | 393249 | -41.91 | 7.25% | D3-J3-5 | 1459 | -37.00 | 2.42% |
| V5-40 | 389736 | -35.24 | 0.01% | D3-J2-1 | 10353 | -28.88 | 6.18% |
| V5-41 | 382229 | -35.24 | 0.01% | D3-J2-2 | 10549 | -25.73 | 11.08% |
| V6-42 | 380394 | -32.46 | 0.00% | D3-J2-3 | 10769 | -24.72 | 13.45% |
| V6-43 | 375660 | -27.82 | 0.08% | D3-J2-4 | 10916 | -31.16 | 6.62% |
| V7-1 | 372720 | -29.73 | 0.30% | D3-J2-5 | 11035 | -25.89 | 5.76% |
| V5-43 | 356146 | -38.79 | 0.03% | D3-J2-6 | 11131 | -23.53 | 0.09% |
| V7-2 | 349368 | -26.29 | 0.59% | D3-J2-7 | 11363 | -29.49 | 19.68% |
| V10 | 337757 | -42.77 | 0.00% | D2-J2-1 | 995 | -28.88 | 12.30% |
| V12-1 | 324869 | -35.81 | 3.55% | D2-J2-2 | 1191 | -25.73 | 18.12% |
| V12-2 | 310231 | -39.96 | 0.08% | D2-J2-3 | 1411 | -24.72 | 26.72% |
| V14 | 307751 | -33.72 | 1.22% | D2-J2-4 | 1558 | -31.16 | 13.26% |
| V15 | 305417 | -33.54 | 2.24% | D2-J2-5 | 1677 | -25.89 | 4.53% |
| ***V16*** | ***300679*** | ***-48.92*** | ***0.09%*** | D2-J2-6 | 1773 | -23.53 | 0.09% |
| V19-1 | 287461 | -31.37 | 2.42% | D2-J2-7 | 2005 | -29.49 | 24.98% |
